# MnO_2_/g-C_3_N_4_/PPy Ternary Composite for Congo Red Adsorption from Water: Preparation and Comparative Performance

**DOI:** 10.3390/ma19194225

**Published:** 2026-10-05

**Authors:** Sami Hummadi, Radi A. Alsulami, Mohammad Omaish Ansari, Ammar Melaibari, Asim Jilani

**Affiliations:** 1 Department of Mechanical Engineering, Faculty of Engineering, King Abdulaziz University, Jeddah 21589, Saudi Arabia; rasalsulami@kau.edu.sa (R.A.A.); aamelaibari@kau.edu.sa (A.M.); 2 Center of Nanotechnology, King Abdulaziz University, Jeddah 21589, Saudi Arabia; moansari@kau.edu.sa; 3 Clean Energy Technologies Research Institute (CETRI), Process Systems Engineering, Faculty of Engineering & Applied Science, University of Regina, 3737 Wascana Parkway, Regina, SK S4S 0A2, Canada; asim.jilani@uregina.ca

**Keywords:** Congo Red, adsorption, wastewater treatment, MnO_2_, g-C_3_N_4_, polypyrrole, ternary composite, dye removal

## Abstract

**Highlights:**

- Single, binary, and ternary adsorbents were compared for Congo Red removal.
- The ternary composite showed the highest observed removal: 75.1% at 120 min.
- Three independent ternary-composite runs achieved approximately 74–75% removal at 120 min.

**Abstract:**

The presence of synthetic dyes in wastewater continues to create problems for water treatment and increases the need for adsorbent materials that can provide effective dye removal. MnO_2_, g-C_3_N_4_, and PPy have been widely studied for pollutant removal, but direct comparisons of their individual, binary, and ternary systems under common adsorption conditions remain limited. In this study, six adsorbents were prepared and compared for Congo Red (CR) removal in the dark. The adsorption experiments were conducted using approximately 40.0 mg of adsorbent in 40 mL of a 200 mg/L CR solution at ambient laboratory temperature. The prepared materials were characterized using XRD, SEM–EDS, XPS, FTIR, nitrogen adsorption–desorption analysis, UV–Vis spectroscopy, and PL spectroscopy to examine their structural, morphological, elemental, surface-chemical, textural, and optical properties. Among the tested samples, the ternary composite showed the highest final Congo Red removal, reaching 75.1% after 120 min, with an observed adsorption capacity $q_{120}$ of 150.20 mg g^−1^. Three independent adsorption experiments also produced close final removal values, supporting the repeatability of the ternary composite. The removal ranking did not follow the BET surface-area ranking, suggesting that surface area alone could not explain the observed performance. Changes in the FTIR spectra after adsorption supported Congo Red removal but did not identify the specific interactions between the dye and the adsorbent surface.

## 1. Introduction

The expansion of industrial and urban activities has increased the amount of untreated wastewater entering aquatic environments, creating environmental and public health concerns in many regions [1,2]. Synthetic dyes are among the important pollutants released from industries such as textile, paper, leather, and printing. Their removal can be difficult because many of these dyes have complex molecular structures, remain chemically stable in water, and resist biological degradation [1,2]. When discharged into natural water bodies, they can reduce water quality and change its appearance. They may also reduce light penetration in water, disrupt aquatic ecosystems, and have toxic, mutagenic, or carcinogenic effects on living organisms [1,2]. For these reasons, there is a continuing need for dye-removal methods that are efficient, economical, and environmentally sustainable.

Congo Red (CR) is an anionic azo dye that is used in textile, paper, rubber, and printing applications because of its strong coloring and chemical stability [3]. The same stability that makes the dye useful in these applications also makes it difficult to biodegrade. Its aromatic structure allows it to remain in aquatic environments for extended periods [3]. Congo Red exposure has also been linked to harmful environmental and health effects. Therefore, removing the dye from industrial wastewater before discharge is an important part of reducing its environmental impact [3].

Several physical, chemical, and biological methods have been investigated for dye removal from wastewater. These include coagulation–flocculation, membrane filtration, advanced oxidation processes, biological degradation, photocatalysis, and adsorption [2,4,5,6]. Although these methods can provide high removal efficiencies, their practical use may be limited by factors such as operating cost, process complexity, membrane fouling, or the generation of secondary pollution [1,2,3]. Adsorption is widely considered a useful alternative because it can be operated relatively simply, generally requires low energy input, and can achieve high removal efficiency in different wastewater systems [5,6]. Its performance, however, is strongly influenced by the physicochemical properties of the adsorbent. This has encouraged continued work on adsorbent materials with higher capacity, better stability, and improved reusability [5].

Recent work has also examined composite adsorbents formed by combining materials with complementary structural and surface properties [5,6,7]. Such hybrid materials can provide multiple adsorption sites, improved surface heterogeneity, and enhanced interactions with dye molecules compared with their individual components, which may improve the overall adsorption performance [5,6,7]. More broadly, a recent study of heterostructured materials for water-treatment applications has shown that engineered interfaces can influence pollutant-removal behavior through interfacial processes [8]. Although the specific mechanism differs from the adsorption system investigated here, this finding highlights the potential functional importance of interfaces in multicomponent materials.

Transition metal oxides and conducting polymers are widely studied as functional adsorbents because of their varied surface chemistries and available active sites for pollutant removal [9,10,11]. Among these materials, manganese dioxide (MnO_2_) is of particular interest because of its low cost, environmental compatibility, surface reactivity, adsorption capacity, and oxidation ability, which allow it to interact with various organic pollutants [9]. In addition to its adsorption capability, MnO_2_ has also been widely explored in photocatalytic and environmental remediation applications because of its favorable surface characteristics and chemical stability [9,12]. However, the adsorption performance of pristine MnO_2_ may be limited by particle aggregation, which can reduce the exposed surface available for pollutant interaction, and this has encouraged its use in composite materials [9].

Another material of interest is graphitic carbon nitride (g-C_3_N_4_), which has a layered graphitic structure, high chemical stability, and a nitrogen-rich framework that provides abundant surface functional groups capable of interacting with organic contaminants [13,14]. These properties have led to its use in photocatalysis and other environmental remediation applications, where both its surface characteristics and electronic structure can influence pollutant removal [4,13]. However, the practical performance of pure g-C_3_N_4_ is often restricted by the limited number of readily accessible adsorption sites and the tendency of nanosheets to restack, reducing the effective surface area available for pollutant adsorption [14,15]. For this reason, g-C_3_N_4_ is frequently combined with other functional materials to improve its adsorption characteristics [14].

Conducting polymers, particularly polypyrrole (PPy), have also been studied as adsorbent materials because of their chemical stability, simple preparation, and conjugated π-electron system, which can promote interactions with aromatic dye molecules through π–π interactions and other intermolecular forces [10,11,16]. Furthermore, the nitrogen-containing functional groups within the PPy backbone provide additional adsorption sites that contribute to enhanced affinity toward various organic pollutants [16]. Despite these advantages, pure PPy may exhibit particle agglomeration and limited mechanical stability during practical application, which can adversely influence its adsorption performance [10,11,16]. Using PPy as part of a composite is therefore one approach that may improve both its adsorption performance and structural stability [16].

Previous studies have examined different combinations of MnO_2_, g-C_3_N_4_, and conducting polymers for environmental applications. MnO_2_/g-C_3_N_4_ composites have been investigated for photocatalytic degradation of organic pollutants because the combination can improve charge separation and catalytic performance under light irradiation [12,17]. Similarly, PPy/g-C_3_N_4_ composites have shown improved pollutant removal owing to the complementary structural and surface properties of both materials [18,19]. Materials containing MnO_2_ together with conducting polymers have also been tested for the removal of metal ions and related contaminants, including U(VI), Eu(III), and Cr(VI) species [20,21,22]. These studies show that combining components can change their surface and functional properties. However, most previous work has focused on photocatalytic degradation or on pollutants other than Congo Red, while direct comparison of the individual materials, binary composites, and ternary systems under the same adsorption conditions remains limited [12,17,18,19,20,21,22].

This gap provides the basis for examining whether combining the three components can improve Congo Red adsorption under simple laboratory conditions without photocatalytic irradiation. Therefore, this study prepared MnO_2_, g-C_3_N_4_, PPy, the MnO_2_/g-C_3_N_4_ and MnO_2_/PPy binary composites, and a ternary MnO_2_/g-C_3_N_4_/PPy composite to evaluate their Congo Red adsorption performance. The samples were tested under identical batch adsorption conditions and were characterized using XRD, SEM–EDS, XPS, FTIR, nitrogen adsorption–desorption analysis, UV–Vis spectroscopy, and PL spectroscopy to relate their adsorption behavior to the structural, morphological, elemental, surface-chemical, textural, and optical properties of the prepared materials. The novelty of the present study lies in the controlled comparison of the single, binary, and ternary MnO_2_/g-C_3_N_4_/PPy systems under identical Congo Red adsorption conditions. This design enables the effect of combining the three components to be evaluated directly, while the characterization results provide complementary information on the changes associated with composite formation. The operating conditions were therefore kept fixed throughout the study to enable a controlled comparison between the prepared materials rather than to optimize the individual adsorption parameters.

## 2. Materials and Methods

### 2.1. Materials and Chemicals

In this study, the chemicals used included potassium permanganate (KMnO_4_, 99–100.5%, puriss., Sigma-Aldrich, St. Louis, MO, USA), hydrochloric acid (HCl, 37%, Honeywell Research Chemicals, Seelze, Germany), urea (CH_4_N_2_O), pyrrole (C_4_H_5_N, 99%, extra pure, Acros Organics, Geel, Belgium), ferric chloride (FeCl_3_, anhydrous, 98%, Extra Pure, Loba Chemie, Pvt. Ltd., Mumbai, India), Congo Red indicator (CR, laboratory reagent grade, Techno Pharmchem, Bahadurgarh, Haryana, India), ethanol, and deionized water. KMnO_4_, urea, pyrrole, and FeCl_3_ served as the starting materials for preparing MnO_2_, g-C_3_N_4_, PPy, and their composites. Congo Red was selected as the model pollutant, whereas ethanol and deionized water were used during washing, dilution, and solution preparation. No further purification of the chemicals was carried out unless otherwise stated.

### 2.2. Preparation of MnO_2_ Nanostructures

MnO_2_ nanostructures were prepared using a hydrothermal method, which is commonly used for MnO_2_-based materials [12]. First, 1.975 g of KMnO_4_ was dissolved in deionized water inside a Teflon-lined autoclave vessel. Then, 4.5 mL of concentrated HCl (37%) was added dropwise while stirring continuously. After 15 min of stirring, the solution turned dark purple. The autoclave was sealed and maintained in an oven at 140 °C for 24 h. Once cooled to room temperature, the black solid was recovered, rinsed several times with ethanol and deionized water, and dried overnight. The resulting black powder was used as the MnO_2_ sample.

### 2.3. Preparation of Graphitic Carbon Nitride (g-C_3_N_4_)

Graphitic carbon nitride (g-C_3_N_4_) was prepared by thermal polymerization of urea, following the common thermal method used for carbon nitride synthesis [13,23]. About 30 g of urea was loaded into a covered crucible. The crucible was then placed in a muffle furnace, where the temperature was raised at a rate of 5 °C/min to 550 °C and maintained for 4 h. After cooling to room temperature, the resulting pale-yellow solid was collected and ground into a fine powder.

### 2.4. Preparation of Polypyrrole (PPy)

Polypyrrole (PPy) was synthesized by aqueous oxidative polymerization of pyrrole, which is a standard method for preparing conducting PPy-based materials [10,11]. In a typical procedure, 2 mL of pyrrole monomer (1.92 g, density = 0.96 g/mL) was added dropwise to 200 mL of deionized water while stirring continuously. Then, 4.67 g of FeCl_3_ was slowly added as the oxidizing agent at an equimolar ratio. The mixture was stirred for several hours at room temperature until a black precipitate formed, indicating pyrrole polymerization. The formed black precipitate was separated from the mixture and rinsed repeatedly with ethanol and deionized water. It was then dried at 60 °C to obtain the PPy powder.

### 2.5. Preparation of Binary and Ternary Composites

The MnO_2_/g-C_3_N_4_ composite was prepared by physically combining the two components, following the general approach used in related MnO_2_/g-C_3_N_4_ composite systems [12,17]. Equal masses of MnO_2_ and g-C_3_N_4_ (0.384 g each) were dispersed in 45 mL of ethanol. The suspension was magnetically stirred for 1 h to improve wetting and reduce agglomeration. After stirring, the suspension was kept in an oven at 60 °C for 24 h until dry. A greyish-yellow powder was obtained, indicating the presence of g-C_3_N_4_ in the composite. The MnO_2_/PPy composite was prepared by oxidative polymerization of pyrrole in the presence of MnO_2_, which is a common route for forming PPy-based composite materials [24]. First, 2 mL of pyrrole monomer (1.92 g) was added to 200 mL of deionized water and stirred continuously at room temperature. Then, 4.64 g of FeCl_3_ was added as the oxidizing agent at an equimolar ratio to initiate polymerization. After 15 min of partial polymerization, 0.768 g of MnO_2_ powder, corresponding to 40% of the pyrrole mass, was added to the mixture to allow incorporation and dispersion within the PPy phase. Stirring continued overnight at room temperature. The black product was then recovered and rinsed repeatedly with ethanol and deionized water before being oven-dried at 60 °C. The ternary MnO_2_/g-C_3_N_4_/PPy composite was prepared by sequential addition during the oxidative polymerization of pyrrole, following the general route used for PPy-based composites [10,11]. First, 2 mL of pyrrole monomer (1.92 g) was added to 200 mL of deionized water under continuous stirring. Then, an equimolar amount of FeCl_3_ (4.64 g) was gradually added to initiate polymerization. After 15 min of partial polymerization, 0.384 g of g-C_3_N_4_, corresponding to 20% of the pyrrole mass, was added to the reaction mixture. After another 15 min of stirring, 0.384 g of MnO_2_, also corresponding to 20% of the pyrrole mass, was added. The mixture was stirred overnight at room temperature to improve dispersion and complete polymerization. The final black solid was recovered and rinsed repeatedly with ethanol and deionized water, then dried at 60 °C to give the MnO_2_/g-C_3_N_4_/PPy composite powder.

### 2.6. Characterization Techniques

Several characterization techniques were used to examine different properties of the prepared samples, including their structure, morphology, elemental composition, optical behavior, and surface properties. XRD measurements were obtained using a Rigaku Ultima IV X-ray diffractometer (Rigaku Corporation, Akishima, Tokyo, Japan). The patterns were used to identify the main crystalline features of MnO_2_ and g-C_3_N_4_ and to compare the structural changes observed after the materials were combined. The XRD-derived structural parameters were calculated only for samples exhibiting sufficiently defined crystalline diffraction peaks, using the corresponding peak positions and full width at half maximum (FWHM) values. The apparent crystallite size (*D*) was estimated using the Debye–Scherrer equation, while the microstrain (ε) and dislocation density (δ) were calculated using the following relationships [25]:

(1) $$D=\frac{K\lambda }{\beta cos\theta }$$

(2) $$\varepsilon =\frac{\beta }{4tan\theta }$$

(3) $$\delta =\frac{1}{D^{2}}$$

In these equations, K = 0.9 represents the shape factor, while λ = 0.15406 nm corresponds to the wavelength of Cu Kα radiation. The measured FWHM is represented by β and was expressed in radians, whereas *θ* denotes the Bragg angle. No instrumental broadening correction was applied, and the measured FWHM values were therefore used directly in the calculations. For samples with several selected diffraction peaks, the parameters were calculated separately for each peak, and the mean value was reported. Surface morphology and elemental composition were analyzed with a JEOL JSM-7600F field emission scanning electron microscope (FESEM, JEOL Ltd., Tokyo, Japan) fitted with an energy-dispersive X-ray spectroscopy (EDS) detector. FESEM images were used to compare the morphology and microstructure of the samples, while EDS provided information on their elemental composition and the distribution of the main detected elements within the composites. UV–Vis measurements were carried out with a PerkinElmer Lambda 750 UV/VIS/NIR spectrometer (PerkinElmer, Shelton, CT, USA). The measurements were used both to examine the optical absorption of the prepared materials and to follow changes in Congo Red absorbance during the adsorption tests. PL spectra were recorded using a Shimadzu RF-5301PC spectrofluorophotometer (Shimadzu Corporation, Kyoto, Japan). The spectra were compared to observe differences in optical emission between the individual materials and the composites and to support the discussion of possible electronic interactions after composite formation. XPS measurements were performed using a PHI 5000 VersaProbe II scanning XPS microprobe (ULVAC-PHI, Inc., Chigasaki, Kanagawa, Japan). Survey spectra were collected for all prepared samples, while high-resolution C 1s, N 1s, and O 1s spectra were obtained for the MnO_2_/g-C_3_N_4_/PPy composite. Peak fitting was performed using a Shirley background and Gaussian components, and the main C 1s peak was referenced to 284.8 eV. FTIR spectra were recorded using a Thermo Scientific Nicolet iS10 spectrometer equipped with a Smart iTX ATR accessory (Thermo Fisher Scientific, Waltham, MA, USA). The MnO_2_/PPy, MnO_2_/g-C_3_N_4_, and MnO_2_/g-C_3_N_4_/PPy composites were analyzed before and after Congo Red adsorption over 400–4000 cm^−1^ using 16 scans at a resolution of 4 cm^−1^. Nitrogen adsorption–desorption measurements were performed at 77 K using a NOVA 1200e surface area and pore size analyzer (Quantachrome Instruments, Boynton Beach, FL, USA). The samples were degassed at 100 °C for 3 h before analysis. The surface area was calculated using the multipoint BET method, while the pore-size distribution, pore volume, and DFT surface area were determined using NLDFT.

### 2.7. Batch Adsorption Experiments

**Figure 1:**
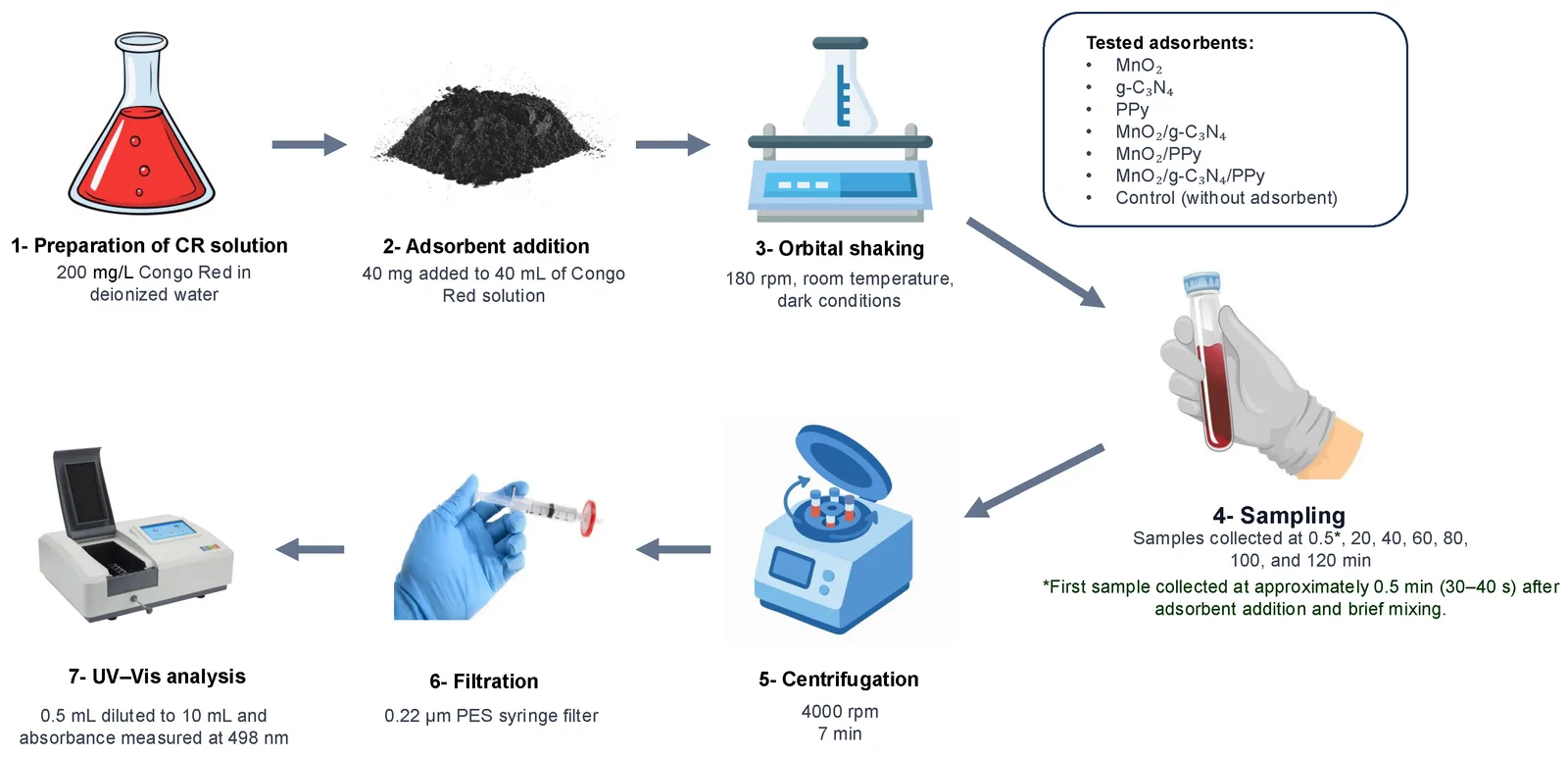
Schematic representation of the batch adsorption procedure used for Congo Red removal. * First sample collected at approximately 0.5 min (30–40 s) after adsorbent addition and brief mixing.

All prepared adsorbents were tested under the same batch conditions so that their Congo Red adsorption behavior could be compared directly. Congo Red (CR) was used as the model dye pollutant. For each experiment, a fresh aqueous CR solution with an initial concentration of 200 mg/L was prepared using deionized water. Before adding any adsorbent, an initial reference sample was collected from the freshly prepared CR solution, and its absorbance was measured to establish the initial reference absorbance $A_{0}$ corresponding to an initial dye concentration $C_{0}$ of 200 mg/L. This pre-contact reference sample contained no adsorbent and therefore did not require adsorbent separation or filtration. Since direct measurement of the 200 mg/L dye solution could exceed the reliable UV–Vis measurement range, 0.5 mL of the sample was diluted with 9.5 mL of deionized water to a final volume of 10 mL before UV–Vis analysis. The resulting dilution was analyzed at 498 nm, which was used as the monitoring wavelength for CR in this study. For each run, approximately 40.0 mg of adsorbent, including MnO_2_, g-C_3_N_4_, PPy, the binary composites, or the ternary composite, was added to 40 mL of the 200 mg/L CR solution in a clean glass beaker. Each beaker was kept covered during the experiment to limit evaporation and contamination. Mixing was carried out using an orbital shaker set to 180 rpm at ambient laboratory temperature (approximately 20–22 °C), without thermostatic temperature control. The experiments were conducted without pH adjustment. The exact initial pH of the original adsorption batches was not recorded. After adsorbent addition and brief mixing, the first post-contact sample was collected after approximately 0.5 min (typically within 30–40 s). Subsequent aliquots of approximately 3.0 mL were withdrawn at 20, 40, 60, 80, 100, and 120 min. The initial pre-contact reference described above was used to establish $A_{0}$ and $C_{0}$ and was not treated as a post-contact adsorption time point. For consistency between measurements, each aliquot was taken from approximately the same position in the beaker after stopping the shaker for less than one minute. Separation of the adsorbent from the liquid was carried out in two steps. Samples were first centrifuged at up to 4000 rpm for 7 min, after which the clear supernatant was collected without disturbing the settled material. Any remaining fine particles were removed by passing the supernatant through a 0.22 µm PES syringe filter. This separation procedure was applied consistently to all samples and time points to reduce scattering effects during UV–Vis measurements. For UV–Vis analysis, 0.5 mL of the filtered sample was diluted to 10 mL using deionized water and then transferred to a cuvette for measurement at 498 nm. The UV–Vis spectrophotometer was blanked using deionized water under the same measurement conditions. A no-adsorbent CR control was also tested using the same sampling, filtration, dilution, and UV–Vis measurement procedure. For the control experiment, the initial reference sample was measured before filtration, whereas the subsequent samples were subjected to the same 0.22 µm filtration procedure used for the adsorption samples. Only minor variations were observed relative to the initial reference, with no appreciable systematic decrease in the measured CR concentration after filtration. This indicates that the filtration procedure did not cause substantial Congo Red loss under the applied analytical conditions. The absorbance results were then used to compare the adsorption behavior of the individual materials, binary composites, and ternary composite. For post-adsorption FTIR analysis, the binary and ternary composites were subjected to the same adsorption conditions described above. The solids were recovered by centrifugation, transferred using a small amount of deionized water, and dried at 60 °C for 10 h prior to FTIR analysis. A schematic representation of the batch adsorption procedure is shown in Figure 1.

### 2.8. Adsorption Calculations and Repeatability

Two parameters were used to compare the adsorption results: removal efficiency (%) and the observed adsorption capacity at 120 min $q_{120}$ (mg/g). Removal efficiency was obtained from the change in CR absorbance measured at 498 nm using the following equation:

(4) $$Removal\left(\%\right)=\frac{\left(A_{0}-\;A_{t}\right)}{A_{0}}\times 100$$

In Equation (4), $A_{0}$ represents the absorbance of the diluted CR solution measured at 498 nm before adsorbent addition, whereas $A_{t}$ represents the absorbance measured at the same wavelength at time t. Since no light-assisted experiments and no degradation analysis were performed in this study, the reported removal values refer only to adsorption removal under the stated conditions. For an additional comparison between the samples, the final removal values were also expressed as an observed adsorption capacity at 120 min. This value represents the amount of Congo Red removed per gram of adsorbent under the conditions used in this study and allows the results to be compared on a mass-of-adsorbent basis. The observed adsorption capacity at 120 min $q_{120}(mg/g)$ was obtained using the following mass-balance relation:

(5) $$q_{120}=\left(C_{0}-C_{120}\right)V/m$$

Here, $C_{0}$ denotes the starting Congo Red concentration and $C_{120}$ is the concentration measured after 120 min, while V and m represent the solution volume and adsorbent mass, respectively. Since the removal efficiency in Equation (4) is reported as a percentage, the fractional removal at 120 min, $R_{120}$, was obtained by dividing the corresponding removal percentage by 100. The observed adsorption capacity at 120 min can therefore be expressed as:

(6) $$q_{120}=\frac{\left(C_{0}R_{120}\right)\;V}{m}$$

Under the nominal experimental conditions: ${(C}_{0}=200\frac{mg}{l},V=40\;ml=0.04\;L,m\approx 40\;mg\approx 0.04\;g)$, the relation can be approximated as:

(7) $$q_{120}\approx 200\times R_{120}\;\mathrm{mg}/g.$$

The comparative adsorption experiments for the individual adsorbents and binary composites were performed as single runs under the same operating conditions. The adsorption experiment for the ternary MnO_2_/g-C_3_N_4_/PPy composite was additionally repeated independently three times to evaluate the repeatability of its adsorption behavior. The three independent runs were conducted under the same operating conditions, and their removal efficiency values were compared to assess the consistency of the measured performance.

## 3. Results and Discussion

### 3.1. Structural Analysis by XRD

**Figure 2:**
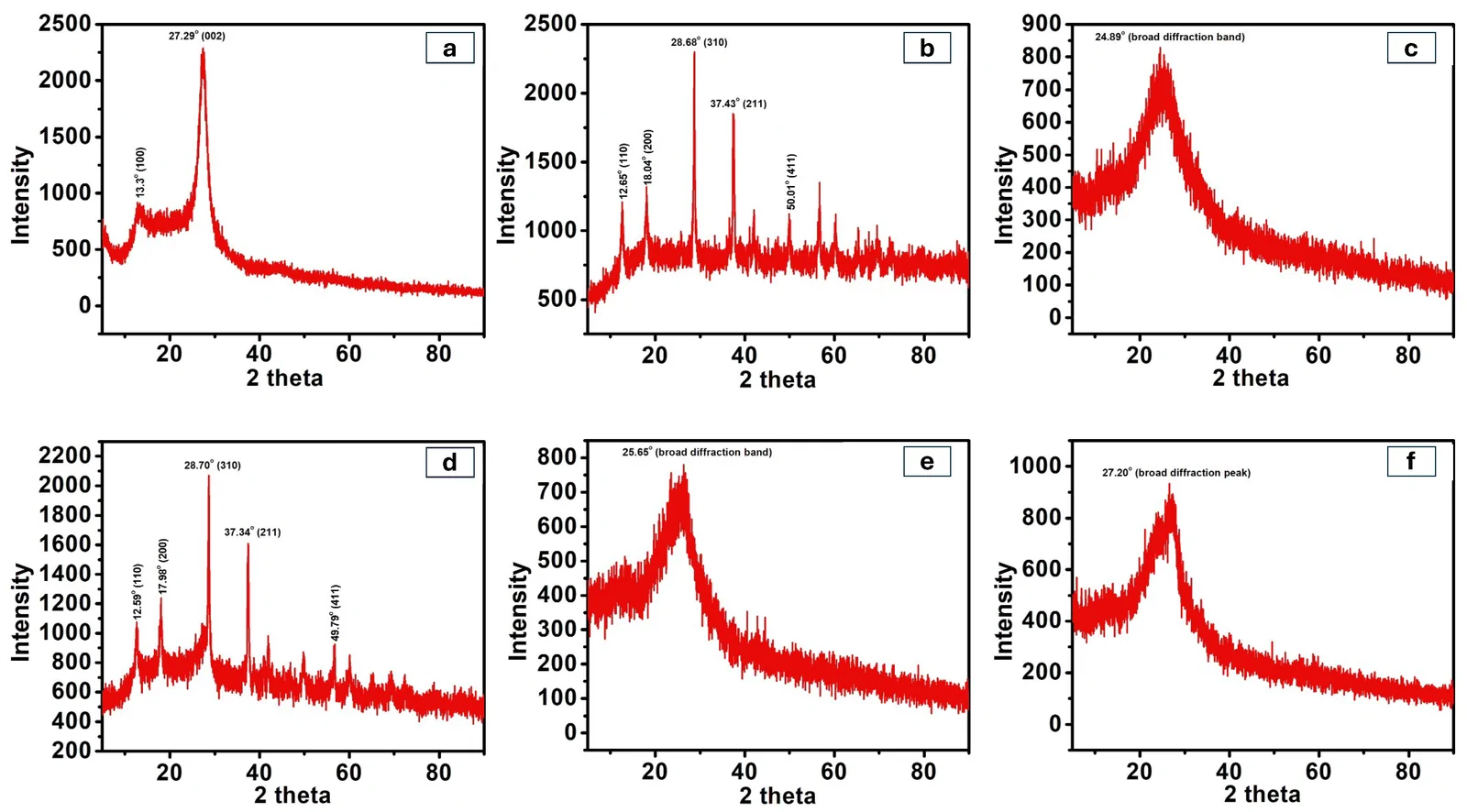
XRD patterns of (**a**) g-C_3_N_4_, (**b**) MnO_2_, (**c**) PPy, (**d**) MnO_2_/g-C_3_N_4_, (**e**) MnO_2_/PPy, and (**f**) MnO_2_/g-C_3_N_4_/PPy, showing the selected 2θ peak positions and their corresponding crystallographic planes (hkl).

The XRD patterns were examined to compare the structural features of the individual materials and the changes observed after composite formation. The selected peak positions were assigned to their corresponding crystallographic planes, denoted as (hkl), the standard labels used to identify different planes within a crystal structure. These assignments were made by comparing the measured 2θ values with published reference XRD patterns for the same materials [13,26,27]. As shown in Figure 2, the g-C_3_N_4_ pattern shows two main diffraction peaks at 2θ = 13.30° and 27.39°. These peaks correspond to the (100) and (002) planes, respectively. Similar peaks near 13° and 27° have been reported for g-C_3_N_4_, with the (100) reflection associated with structural ordering within the layers and the (002) reflection with interlayer stacking [13]. The MnO_2_ sample shows sharper peaks at 12.65°, 18.04°, 28.68°, 37.43°, and 50.01°. The peaks were indexed to the (110), (200), (310), (211), and (411) planes, respectively, in agreement with reported α-MnO_2_ data and JCPDS card No. 44-0141 [26]. In contrast, PPy shows a broad diffraction band centered at approximately 24.89° rather than clearly separated peaks. A similar broad feature near 25.8° has been reported for amorphous PPy, supporting the lower structural order observed in the prepared PPy sample [27].

For the MnO_2_/g-C_3_N_4_ binary composite, the main MnO_2_-related peaks remain visible at 12.59°, 17.98°, 28.70°, 37.34°, and 49.79°, corresponding to the (110), (200), (310), (211), and (411) planes, respectively. These positions are similar to those observed for pure MnO_2_, although some changes in peak intensity and broadening are noticed after combining it with g-C_3_N_4_. The peak at 28.70° lies close to the main g-C_3_N_4_ reflection at 27.39°, which means that contributions from both components may overlap in this region. Together, these features are consistent with the presence of g-C_3_N_4_ and MnO_2_ in the composite pattern [12,17]. The MnO_2_/PPy composite exhibits a broad diffraction band centered at approximately 25.65°, while the sharp MnO_2_ peaks become less distinct. This behavior may result from the contribution of the less-ordered PPy phase to the overall diffraction pattern [24,27]. Similarly, the ternary MnO_2_/g-C_3_N_4_/PPy composite shows a broad band centered at approximately 27.20°, with less distinct crystalline peaks compared with pure MnO_2_ and the MnO_2_/g-C_3_N_4_ composite. The broader pattern suggests a change in structural ordering after PPy was introduced into the ternary system. No clear additional peaks related to a new crystalline phase are observed. Overall, the XRD patterns indicate that the components were combined without producing an obvious new crystalline phase.

Table 1 compares the apparent crystallite size, microstrain, and dislocation density calculated from the XRD data. These parameters provide additional information about peak broadening and structural ordering. For samples exhibiting sufficiently defined crystalline diffraction peaks, peak broadening can provide approximate information on coherent crystallite size and structural strain [25,28]. MnO_2_ reveals the largest apparent crystallite size of 21.7001 nm and the lowest microstrain and dislocation density values, which agree with its sharper diffraction peaks. In comparison, g-C_3_N_4_ exhibits a smaller crystallite size and higher microstrain and dislocation density, consistent with its broader diffraction features. The MnO_2_/g-C_3_N_4_ composite shows an intermediate crystallite size of 14.2132 nm, while its microstrain and dislocation density remain relatively low. This agrees with the presence of the main MnO_2_ peaks, although their intensity and broadening are altered after composite formation. Overall, the calculated parameters for the crystalline samples are consistent with the general peak-broadening trends observed in the corresponding diffraction patterns.

### 3.2. Morphological and Elemental Analysis

**Figure 3:**
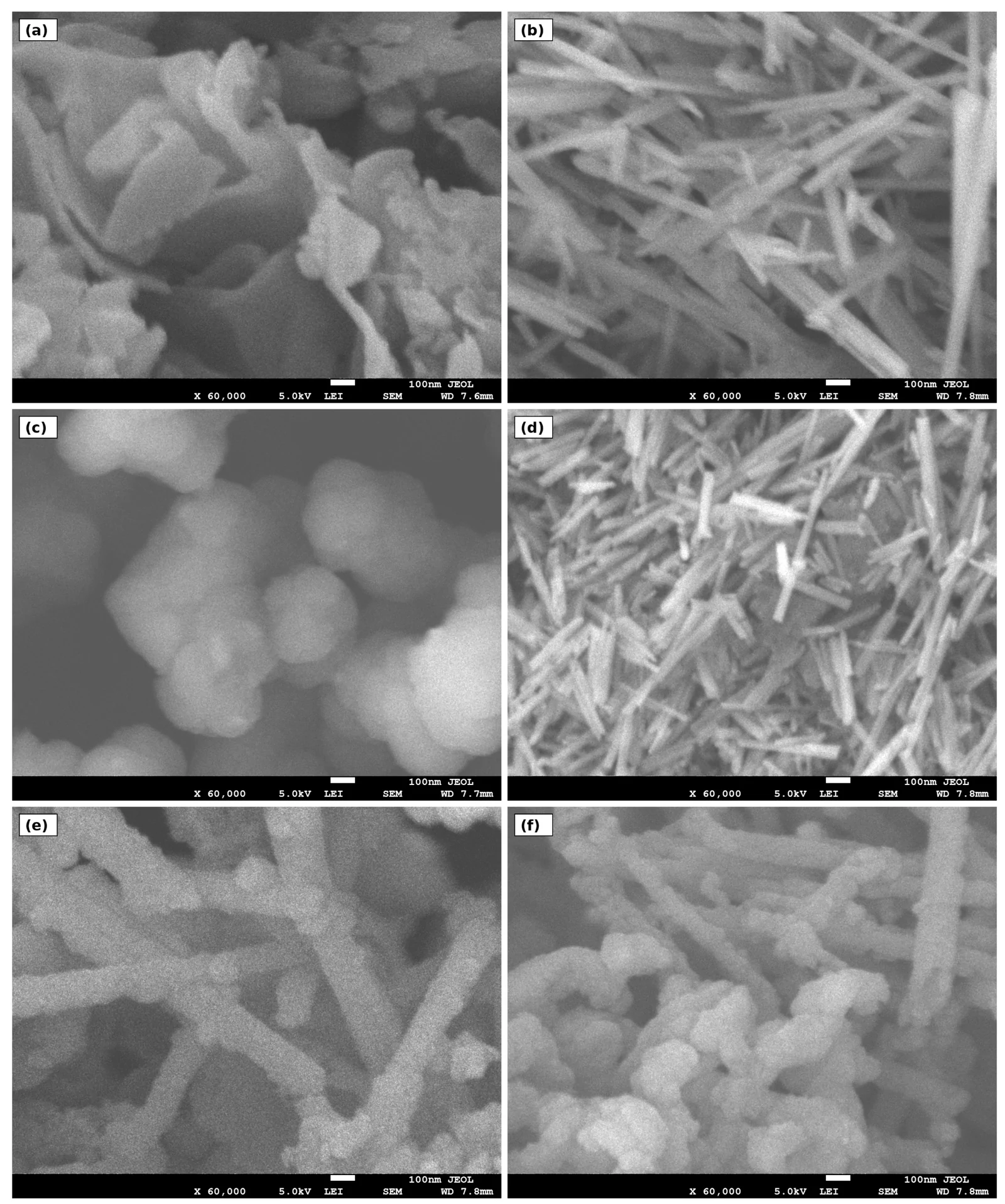
SEM micrographs of the individual materials and composite samples: (**a**) g-C_3_N_4_, (**b**) MnO_2_, (**c**) PPy, (**d**) MnO_2_/g-C_3_N_4_, (**e**) MnO_2_/PPy, and (**f**) MnO_2_/g-C_3_N_4_/PPy.

SEM images were used to compare the surface morphology of the individual materials and their composites. As shown in Figure 3, g-C_3_N_4_ displays irregular layered and block-like particles, while MnO_2_ shows clear rod-like structures. PPy appears as aggregated granular particles with a relatively compact morphology. After composite formation, the surface features become more mixed and less distinct than those of the individual components. In the MnO_2_/g-C_3_N_4_ composite, the rod-like MnO_2_ structures are observed together with the irregular g-C_3_N_4_ phase, showing morphological features from both components. In the MnO_2_/PPy composite, the rod-like structures appear partially covered or surrounded by the polymer phase, giving the surface a rougher, more interconnected morphology. For the ternary MnO_2_/g-C_3_N_4_/PPy composite, both rod-like structures and aggregated polymer-like features are observed within one hybrid structure. Similar morphological features have also been reported for related MnO_2_/g-C_3_N_4_ and MnO_2_/PPy-based materials [12,17,24].

**Figure 4:**
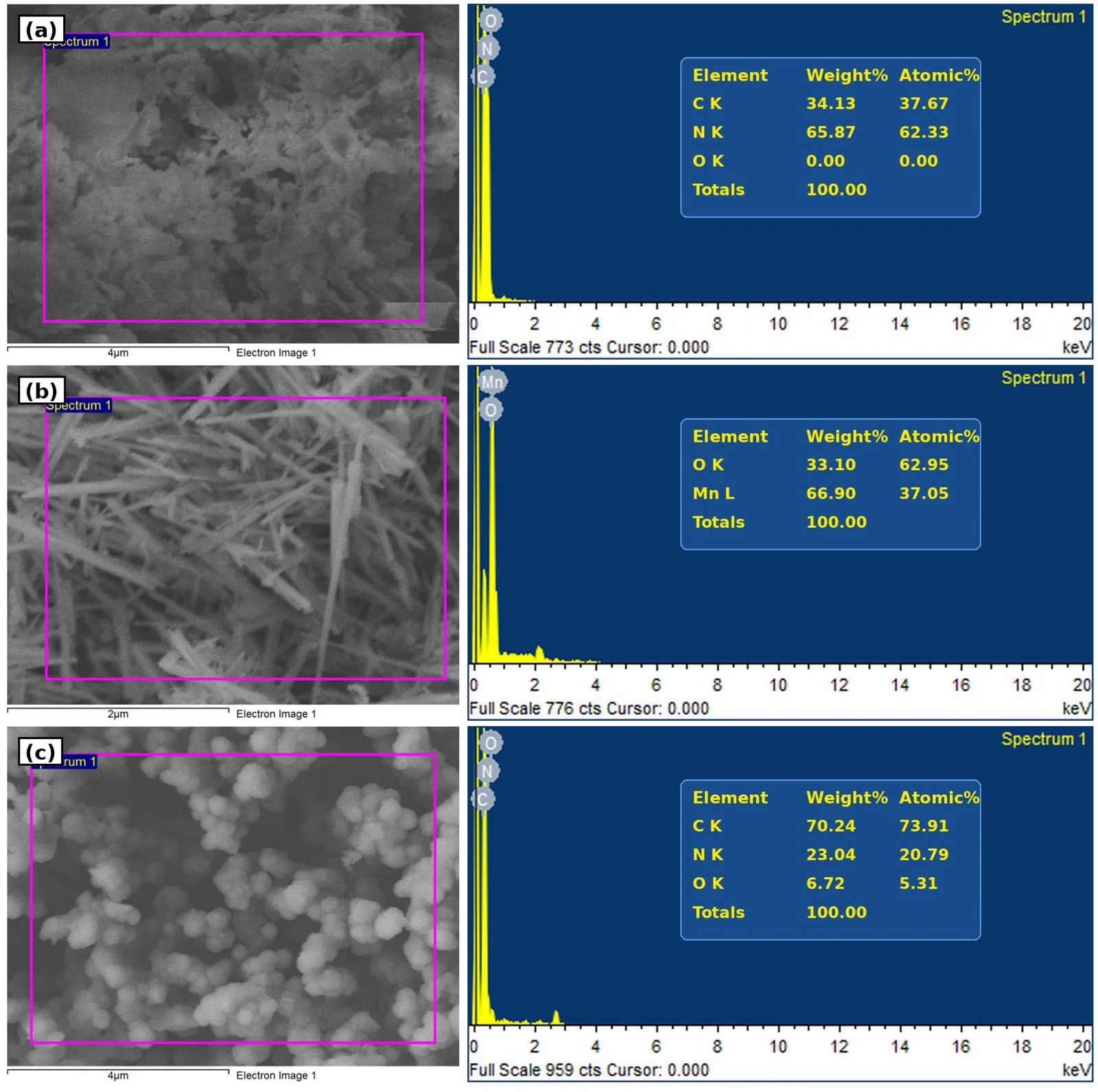
EDS spectra and elemental composition of the individual prepared materials: (**a**) g-C_3_N_4_, (**b**) MnO_2_, and (**c**) PPy. The magenta rectangles indicate the areas selected for EDS analysis, while the yellow traces represent the corresponding EDS spectra.

**Figure 5:**
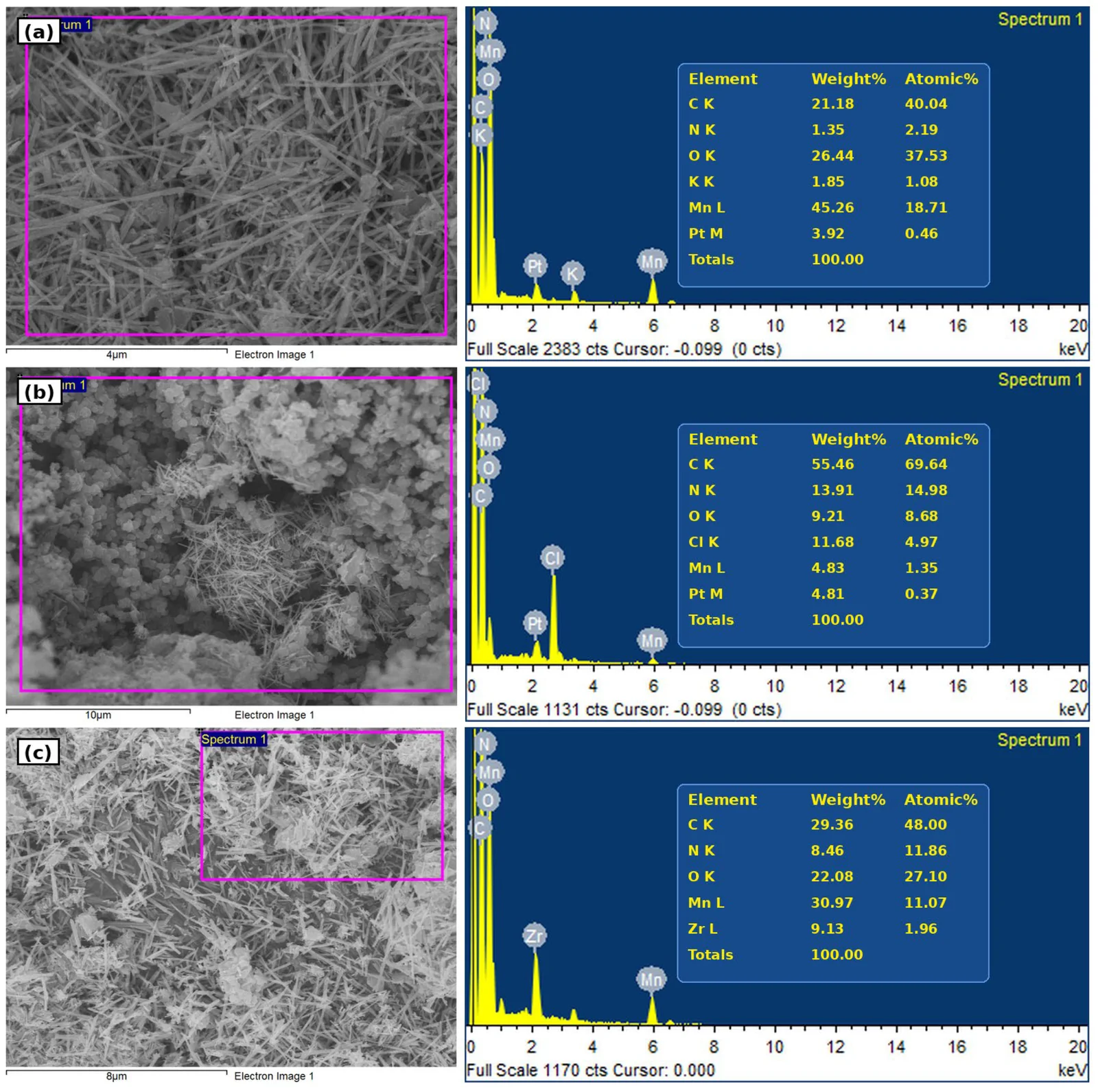
EDS spectra and elemental composition of the prepared composite materials: (**a**) MnO_2_/g-C_3_N_4_, (**b**) MnO_2_/PPy, and (**c**) MnO_2_/g-C_3_N_4_/PPy. The magenta rectangles indicate the areas selected for EDS analysis, while the yellow traces represent the corresponding EDS spectra.

EDS was used to compare the elemental composition of the prepared samples and to check whether the expected elements were present in the composite materials. Figure 4 presents the EDS results of the individual materials, while Figure 5 presents those of the binary and ternary composites. The g-C_3_N_4_ sample mainly shows C and N, while the MnO_2_ sample shows Mn and O. The PPy sample mainly shows C and N, consistent with the chemical structure of PPy. For the composite samples, the EDS spectra show combined elemental signals from the original components. The MnO_2_/g-C_3_N_4_ composite exhibits C, N, Mn, and O signals, while the MnO_2_/PPy composite shows signals related to both PPy and MnO_2_. The ternary MnO_2_/g-C_3_N_4_/PPy composite shows the expected elemental contributions from all three components. Taken together, the SEM and EDS observations are consistent with the presence of the expected components in the prepared binary and ternary materials.

### 3.3. Surface Chemical Analysis by XPS

**Figure 6:**
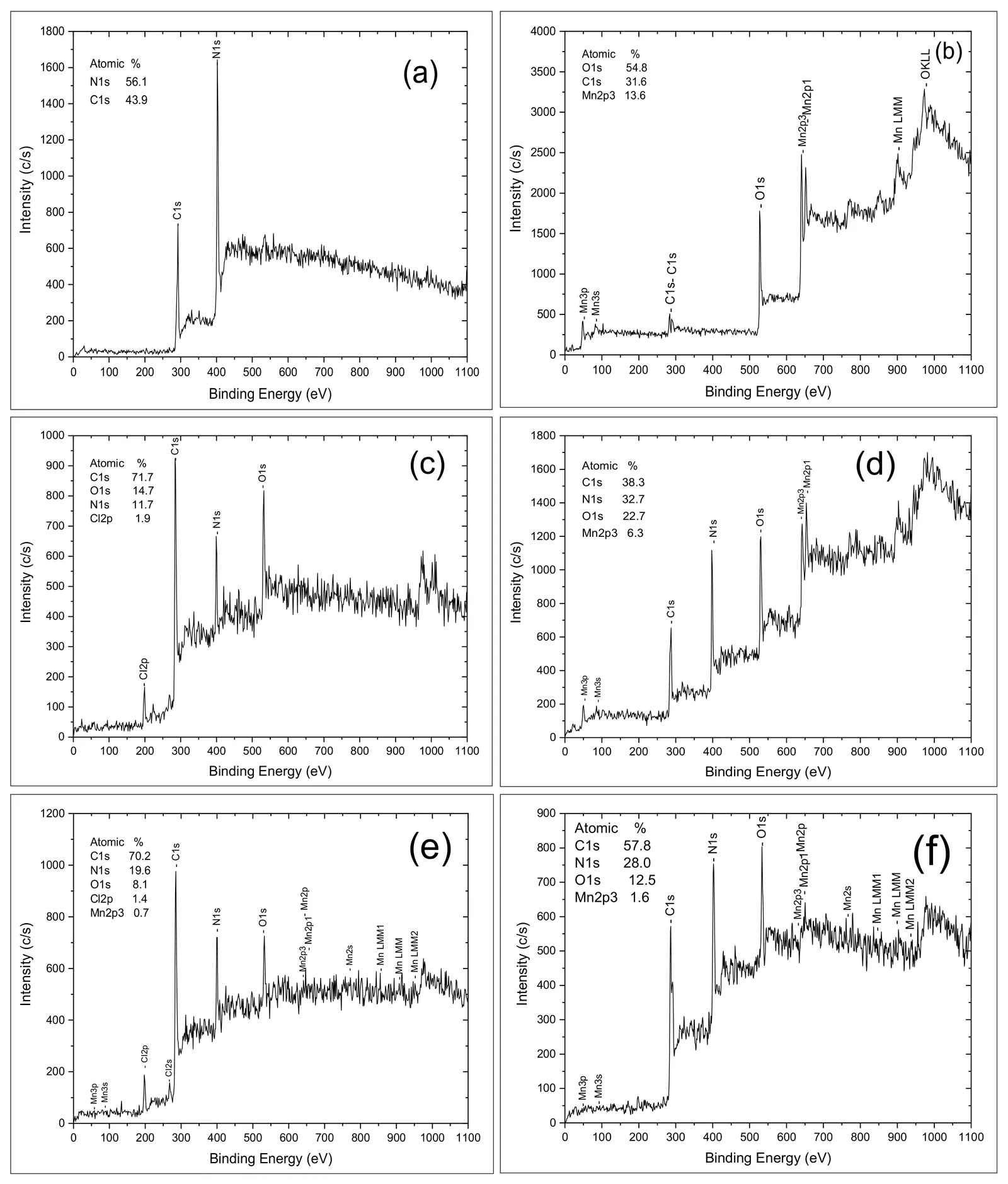
XPS survey spectra of the individual materials and composite samples: (**a**) g-C_3_N_4_, (**b**) MnO_2_, (**c**) PPy, (**d**) MnO_2_/g-C_3_N_4_, (**e**) MnO_2_/PPy, and (**f**) MnO_2_/g-C_3_N_4_/PPy.

XPS survey analysis shown in Figure 6 is used to examine the elemental composition of the surfaces of the prepared materials and composites. The atomic percentages obtained from the survey spectra are summarized in Table 2. The g-C_3_N_4_ sample contains 43.9 at.% C and 56.1 at.% N, which is consistent with the carbon–nitrogen framework of graphitic carbon nitride [13].

The MnO_2_ sample contains 31.6 at.% C, 54.8 at.% O, and 13.6 at.% Mn, with distinct O and Mn signals confirming the presence of manganese oxide species. The PPy sample primarily comprises C, N, and O, with atomic concentrations of 71.7, 11.7, and 14.7 at.%, respectively. After composite formation, the MnO_2_/g-C_3_N_4_ sample contains 38.3 at.% C, 32.7 at.% N, 22.7 at.% O, and 6.3 at.% Mn. The detection of C, N, O, and Mn shows surface contributions from both g-C_3_N_4_ and MnO_2_. Similar XPS survey spectra containing C 1s, N 1s, O 1s, and Mn 2p signals have been reported for MnO_2_/g-C_3_N_4_ composite systems [29]. The MnO_2_/PPy and MnO_2_/g-C_3_N_4_/PPy composites are also dominated by C- and N-containing surface components. Mn was detected at 0.7 at.% in MnO_2_/PPy, whereas the repeat survey of the ternary composite gave 57.8 at.% C, 28.0 at.% N, 12.5 at.% O, and 1.6 at.% Mn. The relatively low Mn surface fraction in the PPy-containing composites may reflect surface coverage and/or differences in component distribution. Cl was quantified in PPy and MnO_2_/PPy at 1.9 and 1.4 at.%, respectively, and may originate from FeCl_3_ used during oxidative polymerization, either as residual chloride-containing species or as chloride counterions associated with oxidized PPy. In the repeat survey measurement of the ternary composite, Cl could not be reliably quantified. Because XPS probes a limited surface region and the composite may exhibit local compositional variation, this result should not be interpreted as confirmation that Cl is completely absent. No Fe signal was detected in the survey spectra, indicating that Fe was below the XPS detection limit at the analyzed surface locations. Accordingly, the survey spectra should be interpreted as local surface-composition measurements and cannot establish the bulk concentrations or spatial distributions of Cl and Fe within the samples.

**Figure 7:**
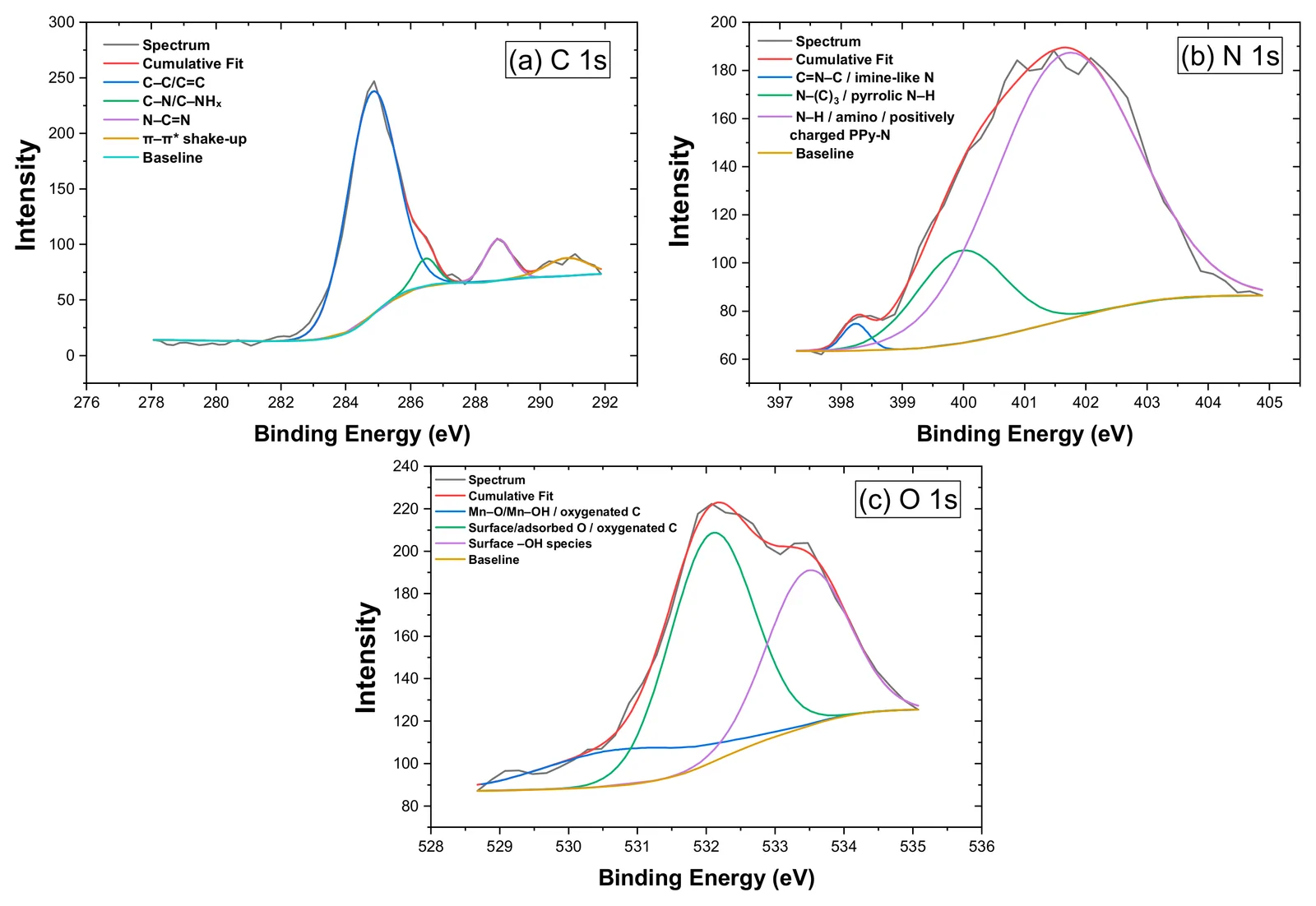
High-resolution XPS spectra and peak deconvolution of the MnO_2_/g-C_3_N_4_/PPy ternary composite: (**a**) C 1s, (**b**) N 1s, and (**c**) O 1s.

To further examine the surface chemical environments of the ternary MnO_2_/g-C_3_N_4_/PPy composite, high-resolution C 1s, N 1s, and O 1s spectra were deconvoluted using a Shirley background and Gaussian components, as shown in Figure 7 and summarized in Table 3. The binding-energy scale was charge-corrected by referencing the main C 1s C–C/C=C component to 284.8 eV. The C 1s spectrum was resolved into components at 284.80, 286.47, 288.70, and 290.82 eV. The components at 284.80 and 286.47 eV are consistent with C–C/C=C and C–N/C–NH_x_ environments, respectively, whereas the 288.70 eV component is characteristic of sp^2^ carbon in N–C=N environments of g-C_3_N_4_ [30]. The higher-binding-energy feature at 290.82 eV is consistent with the π–π* shake-up satellite reported for conjugated PPy structures [31]. The N 1s spectrum contained components at 398.24, 399.96, and 401.68 eV. Because nitrogen environments from g-C_3_N_4_ and PPy overlap substantially within this binding-energy range, the components were interpreted conservatively. The 398.24 eV contribution is consistent with sp^2^ C=N–C nitrogen in g-C_3_N_4_ and/or imine-like nitrogen in PPy, while the 399.96 eV component falls within the reported ranges of bridging N [N–(C)_3_] in g-C_3_N_4_ and neutral pyrrolic N–H in PPy [30,32]. The higher-binding-energy component at 401.68 eV may include contributions from N–H/amino environments and positively charged nitrogen species in PPy [30,32]. The O 1s spectrum was fitted with components at 530.78, 532.08, and 533.47 eV. These binding energies lie within overlapping ranges reported for Mn–O-related oxygen, adsorbed/surface oxygen, hydroxyl-containing species, and oxygenated-carbon environments [31,33]. Therefore, the O 1s components were interpreted conservatively and were not assigned exclusively to a single phase. Overall, the high-resolution XPS results are consistent with the coexistence of surface chemical environments associated with MnO_2_, g-C_3_N_4_, and PPy in the ternary composite.

The survey spectrum also contained weak Mn 3s and Mn 3p contributions. A high-binding-energy feature near 94 eV in the Mn 3s region lies within the range reported for charge-transfer satellite features in manganese oxides [34]. Both Mn 3s multiplet splitting and the position and shape of the Mn 3p photo-line have been used to assess Mn oxidation states by XPS [35]. However, the limited spectral resolution and low signal intensity of the Mn regions in the present survey spectrum did not allow these features to be resolved with sufficient reliability for quantitative oxidation-state analysis. A high-resolution Mn 2p spectrum suitable for reliable fitting was not available. The Mn survey signals were therefore interpreted qualitatively, without determining oxidation-state fractions.

### 3.4. Textural Properties and Pore-Size Distribution

**Figure 8:**
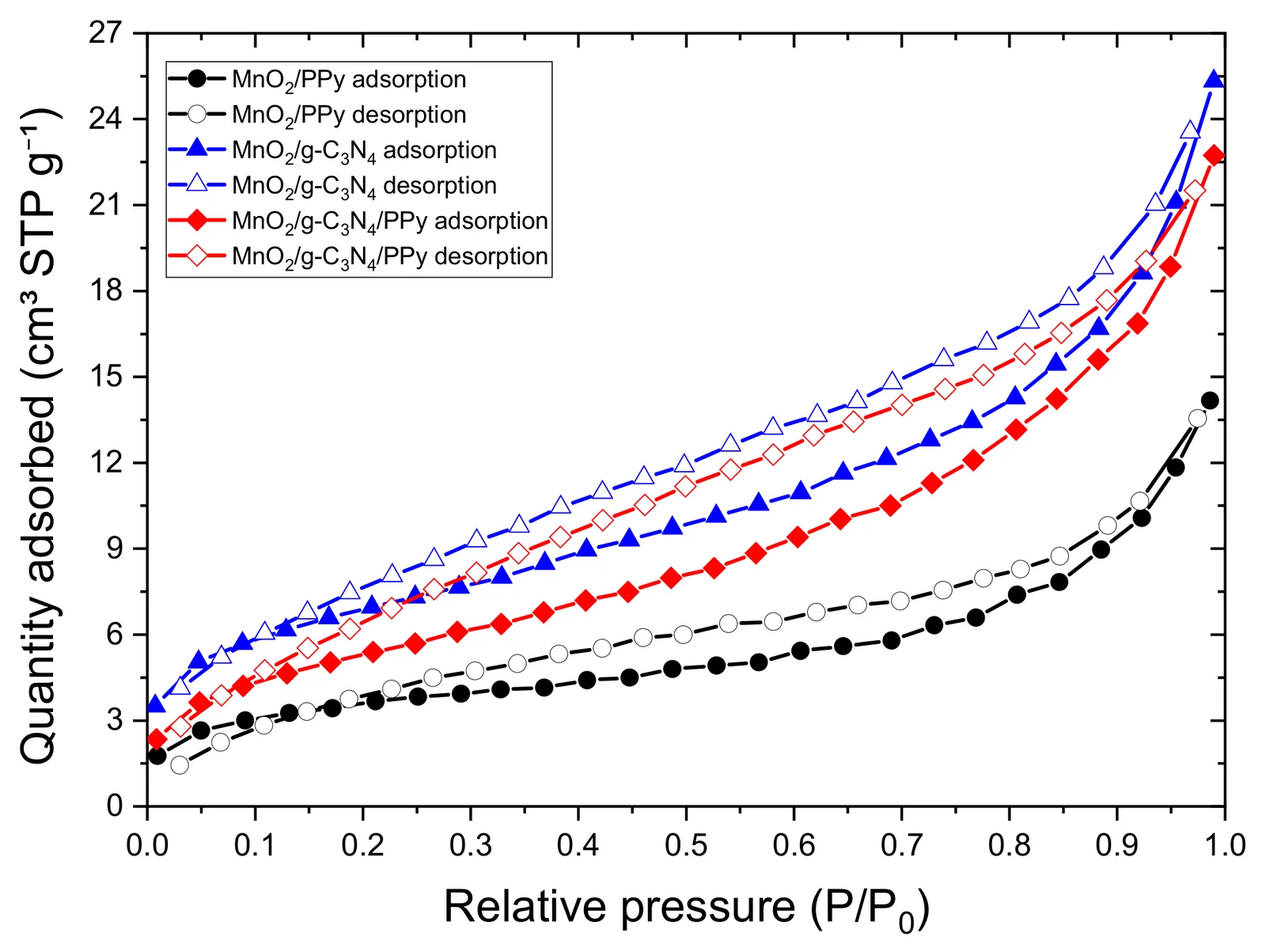
Nitrogen adsorption–desorption isotherms of MnO_2_/PPy, MnO_2_/g-C_3_N_4_, and MnO_2_/g-C_3_N_4_/PPy composites.

The textural properties of the MnO_2_/PPy, MnO_2_/g-C_3_N_4_, and MnO_2_/g-C_3_N_4_/PPy composites were investigated using nitrogen adsorption–desorption analysis. As shown in Figure 8, the amount of nitrogen adsorbed increased progressively with increasing relative pressure for all three composites, with a more pronounced increase as $P/P_{0}$ approached unity. The MnO_2_/g-C_3_N_4_ composite exhibited the highest nitrogen uptake over most of the investigated relative-pressure range, followed by MnO_2_/g-C_3_N_4_/PPy, whereas MnO_2_/PPy showed the lowest uptake. The separation between the adsorption and desorption branches indicates the presence of hysteresis associated with pore condensation and possible network effects in mesoporous structures [36].

**Figure 9:**
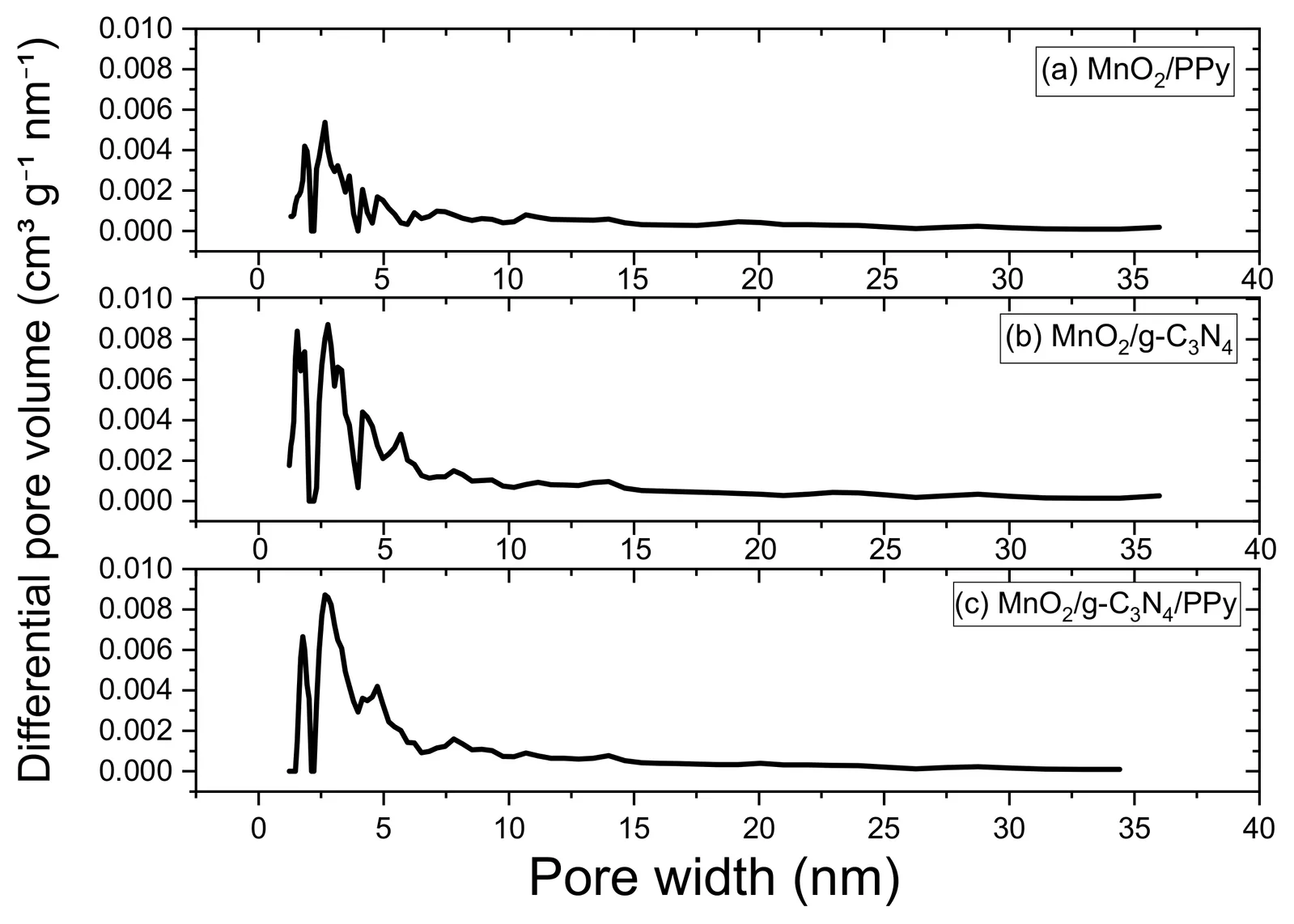
NLDFT pore-size distributions of (**a**) MnO_2_/PPy, (**b**) MnO_2_/g-C_3_N_4_, and (**c**) MnO_2_/g-C_3_N_4_/PPy composites.

The NLDFT pore-size distributions shown in Figure 9 reveal that the most intense contributions occurred within the narrow-mesopore region, particularly at pore widths of approximately 2–6 nm. The modal pore widths were 2.647, 2.769, and 2.647 nm for MnO_2_/PPy, MnO_2_/g-C_3_N_4_, and MnO_2_/g-C_3_N_4_/PPy, respectively. These values fall within the mesopore range of 2–50 nm defined by the International Union of Pure and Applied Chemistry (IUPAC) [36]. Lower-intensity contributions extended toward larger pore widths, indicating that the composites possess broader and non-uniform pore-size distributions rather than a single uniform pore population.

As summarized in Table 4, MnO_2_/g-C_3_N_4_ exhibited the highest BET surface area of 24.203 m^2^ g^−1^, together with the highest NLDFT surface area and pore volume of 17.036 m^2^ g^−1^ and 0.034 cm^3^ g^−1^, respectively. The ternary MnO_2_/g-C_3_N_4_/PPy composite exhibited intermediate values, with a BET surface area of 19.66 m^2^ g^−1^, an NLDFT surface area of 14.045 m^2^ g^−1^, and a pore volume of 0.031 cm^3^ g^−1^. MnO_2_/PPy showed the lowest corresponding values of 12.39 m^2^ g^−1^, 8.370 m^2^ g^−1^, and 0.019 cm^3^ g^−1^, respectively. Notably, the order of Congo Red removal efficiency did not follow the order of the measured textural parameters. After 120 min, the ternary composite achieved the highest removal efficiency of 75.1%, followed by MnO_2_/PPy at approximately 71.3% and MnO_2_/g-C_3_N_4_ at 64.47%, although MnO_2_/g-C_3_N_4_ possessed the highest surface area and pore volume. Therefore, under the investigated conditions, Congo Red adsorption was not governed solely by the BET surface area or pore volume. The chemical nature and accessibility of the surface adsorption sites may also have contributed to the observed performance. Previous studies of PPy-containing adsorbents have reported that nitrogen-containing sites in PPy can participate in the adsorption of anionic Congo Red through interactions such as ion exchange, electrostatic attraction, and π–π interactions [37]. This provides a plausible explanation for the comparatively high removal efficiencies of the PPy-containing composites; however, the present BET results alone do not establish a specific adsorption mechanism.

### 3.5. Optical Properties by UV–Vis and PL

**Figure 10:**
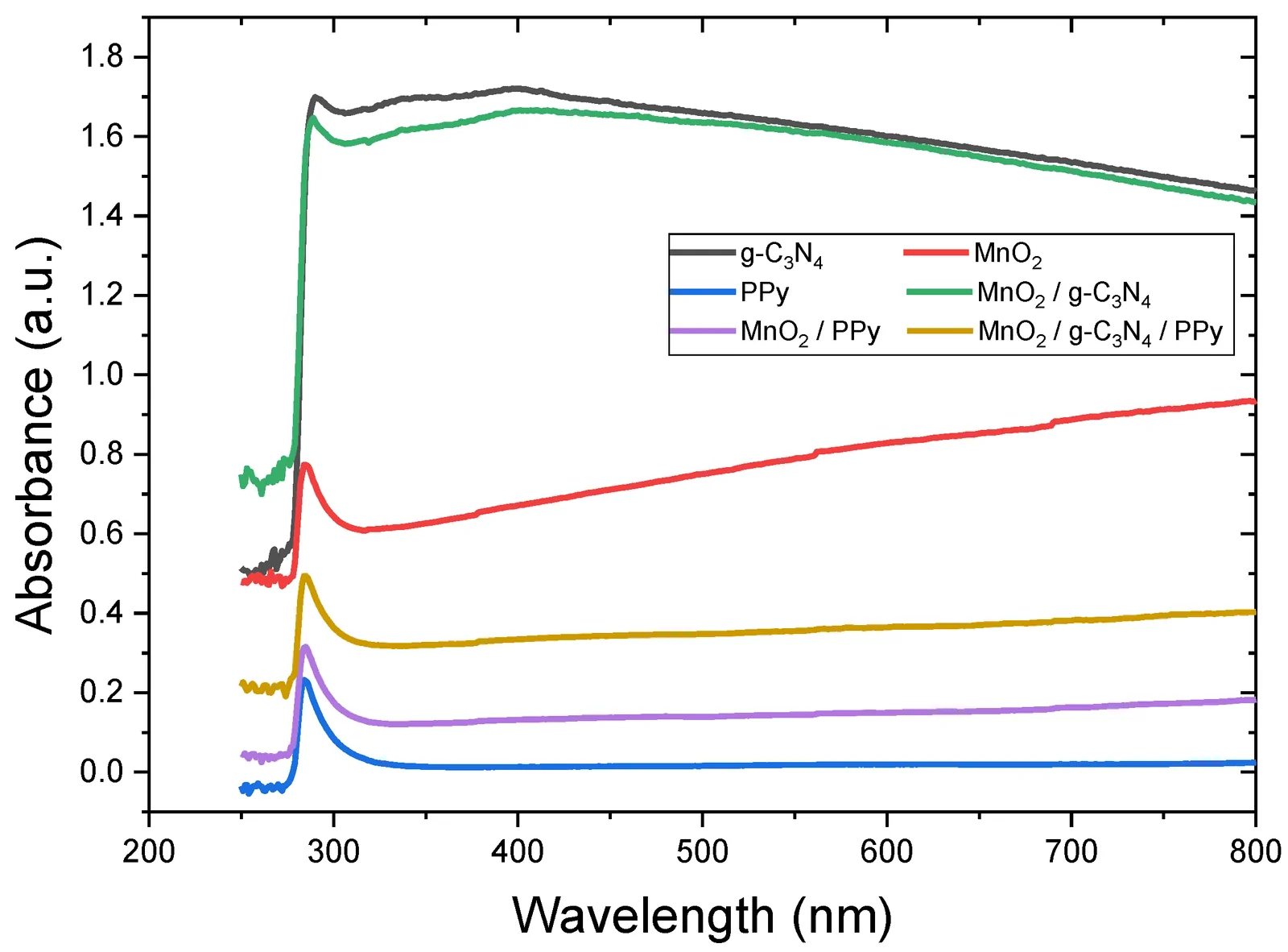
UV-Vis absorbance spectra of all prepared samples.

**Figure 11:**
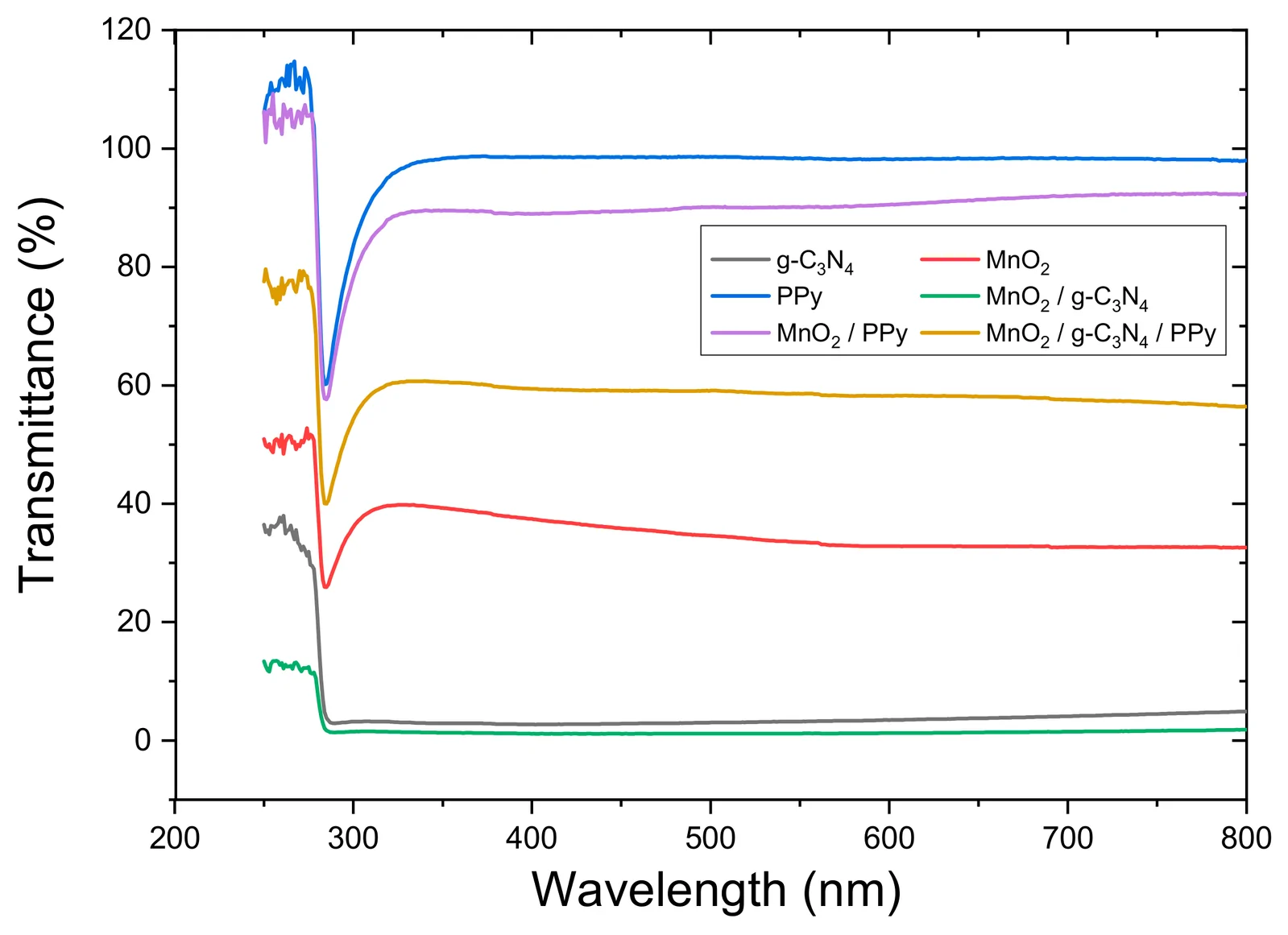
UV-Vis transmittance results for all prepared samples.

UV–Vis spectroscopy was used to compare the optical response of the prepared materials over the range of 250–800 nm. As shown in Figure 10, the spectra differed among the prepared materials, with the composite samples exhibiting optical responses distinct from those of the individual components. The corresponding transmittance spectra in Figure 11 followed the expected inverse trend relative to absorbance. Because the adsorption experiments were performed in the dark, these optical measurements are presented only as supporting material characterization and are not used to explain the adsorption mechanism or to infer photocatalytic activity.

**Figure 12:**
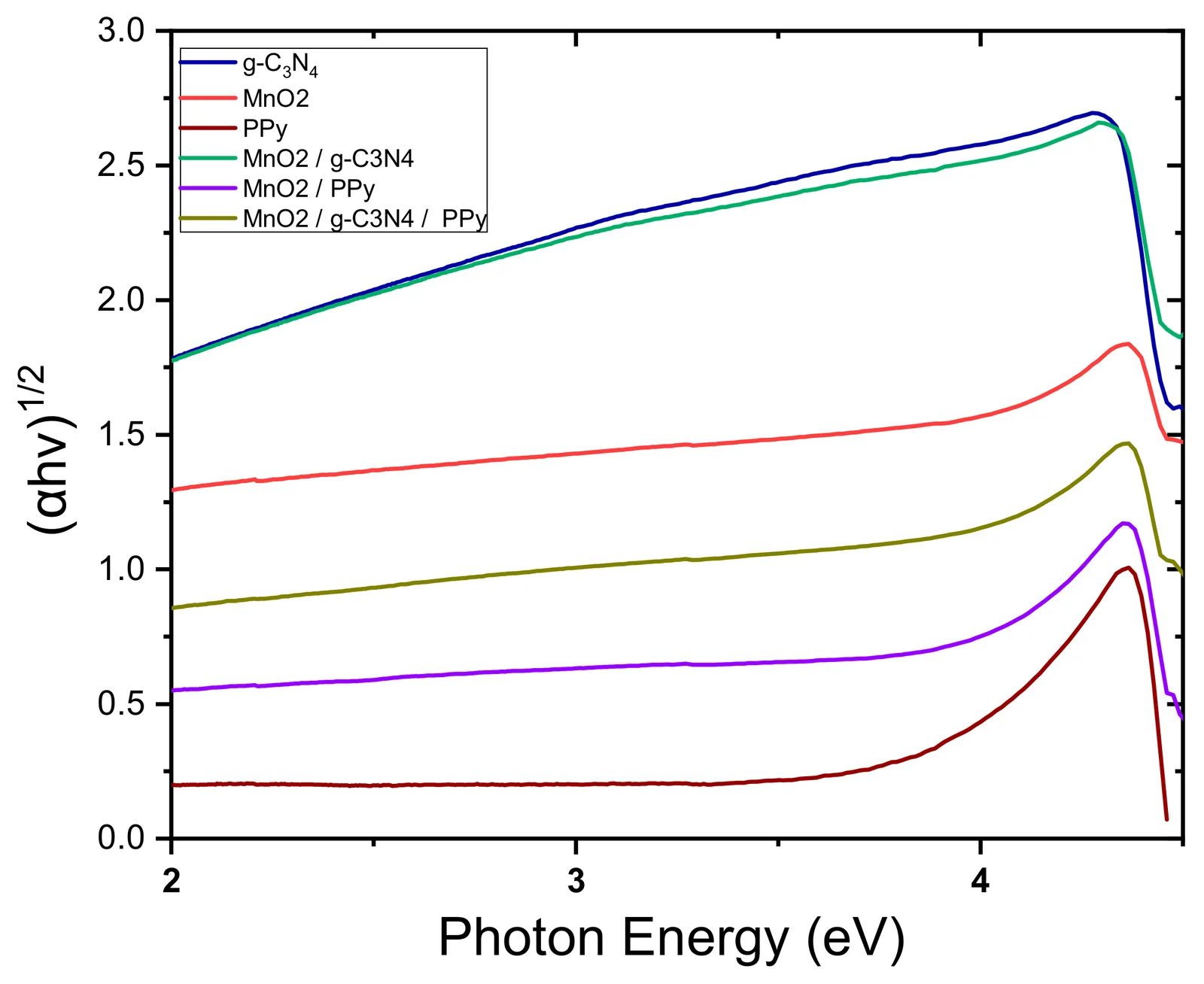
Indirect Tauc-transformed optical response of the prepared samples.

The indirect Tauc-transformed plots of the prepared samples are shown in Figure 12. Differences were observed among the prepared samples, indicating changes in their optical response after composite formation. However, reliable linear extrapolation regions could not be consistently identified for all samples. Since Tauc-derived band-gap estimates can be sensitive to the selection of the linear region and the extrapolation procedure [38]. quantitative band-gap values were not extracted from these plots. The Tauc-transformed data are therefore presented only as comparative optical characterization and are not interpreted as evidence of photocatalytic activity in the present study.

**Figure 13:**
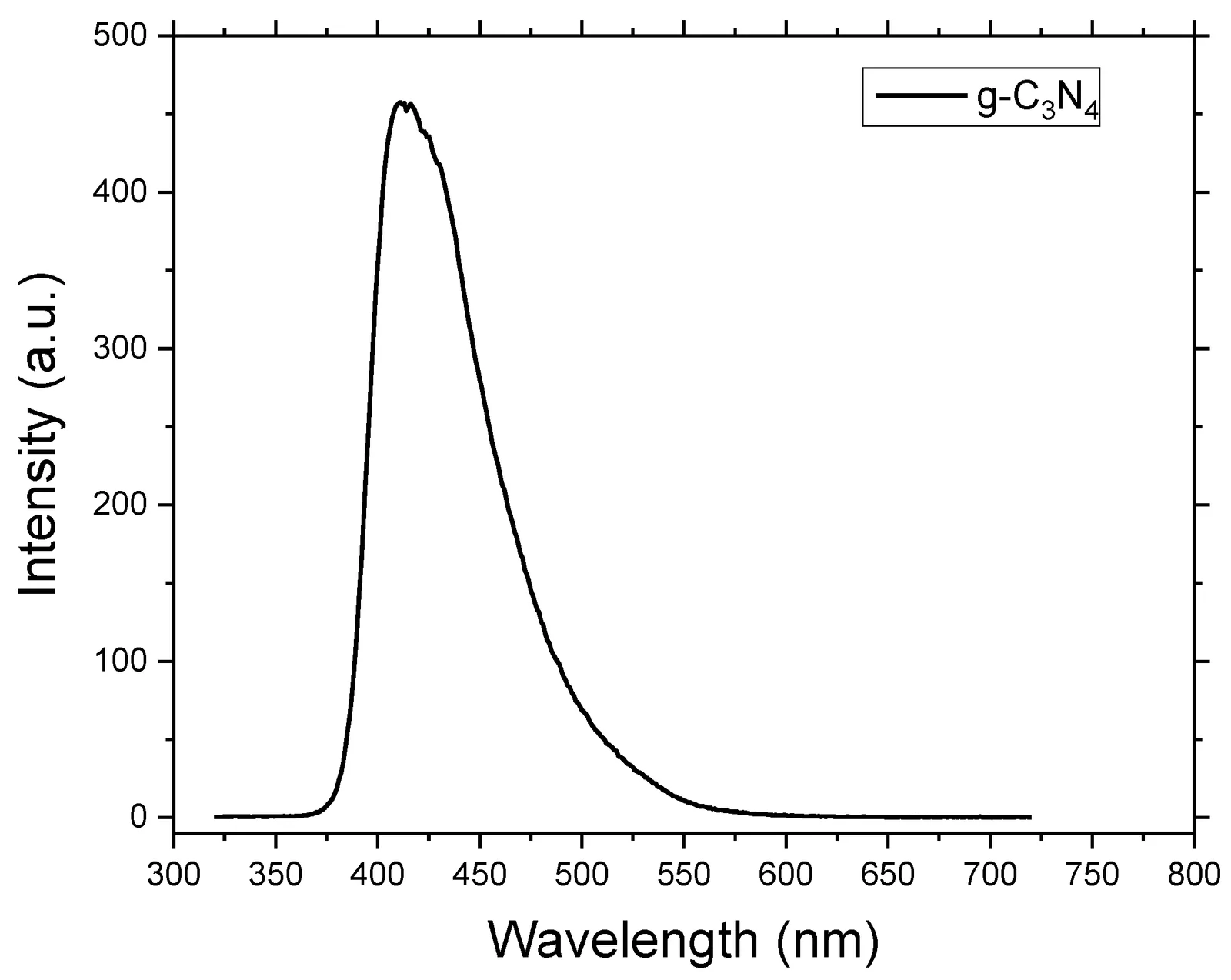
Photoluminescence (PL) spectrum of g-C_3_N_4_.

**Figure 14:**
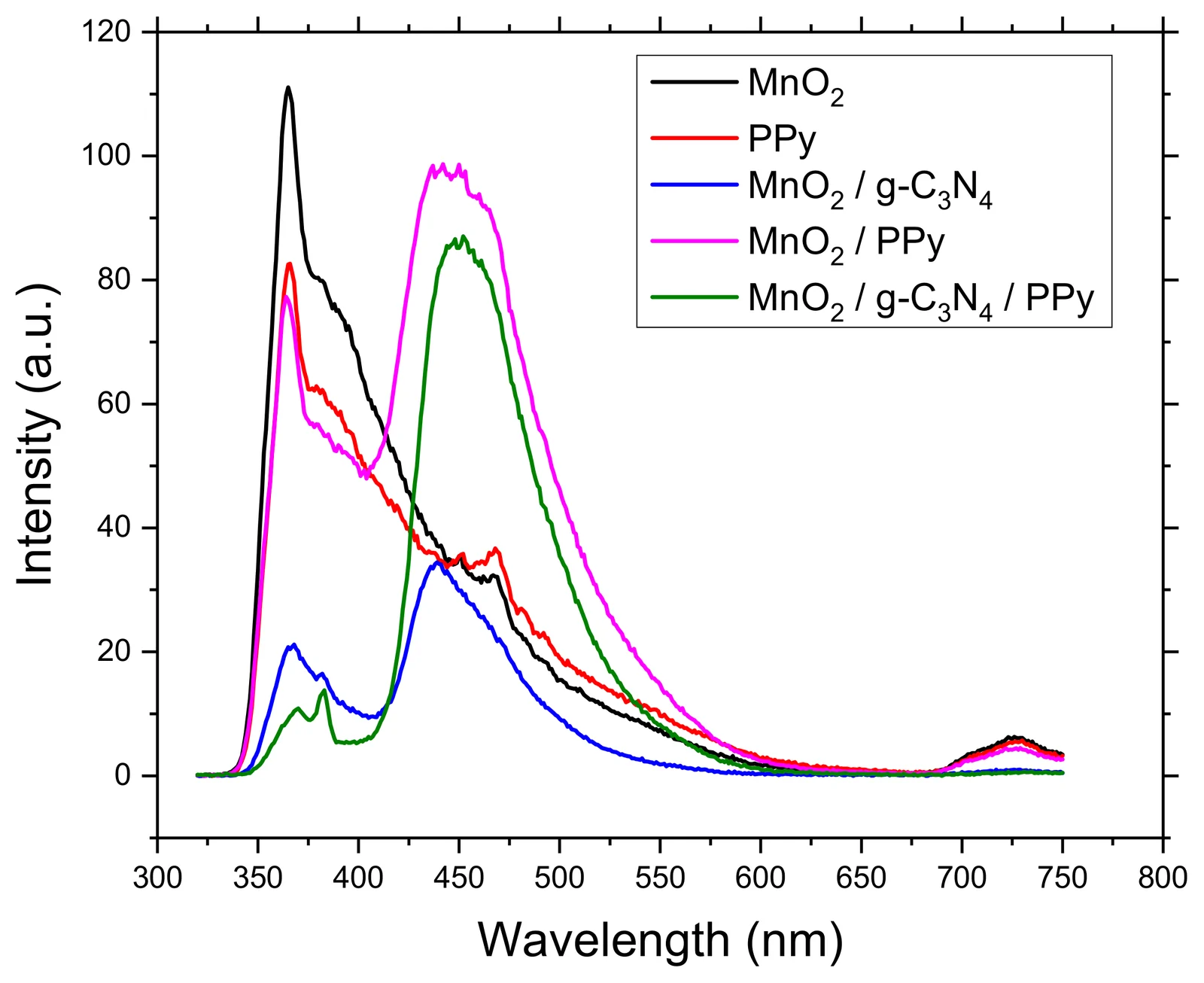
Photoluminescence (PL) spectra of MnO_2_, PPy, MnO_2_/g-C_3_N_4_, MnO_2_/PPy, and MnO_2_/g-C_3_N_4_/PPy.

PL spectra also showed differences in emission intensity and peak shape among the individual and composite materials. The g-C_3_N_4_ sample in Figure 13 exhibited a broad visible emission, whereas the composite samples in Figure 14 showed modified emission profiles after composite formation. Such changes may be consistent with modification of the local electronic environment and interfacial interactions between the constituent materials. A recent heterostructure study has combined PL with complementary techniques such as photoelectrochemical measurements, KPFM, and DFT calculations to evaluate interfacial charge redistribution and charge-transfer behavior [39]. Therefore, although the PL changes observed here indicate that composite formation modified the emission behavior of the materials, PL alone is insufficient to assign a specific charge-transfer pathway. Since the present adsorption experiments were conducted under dark conditions, these results are not interpreted as evidence of photocatalytic activity.

### 3.6. Congo Red Adsorption Performance

**Figure 15:**
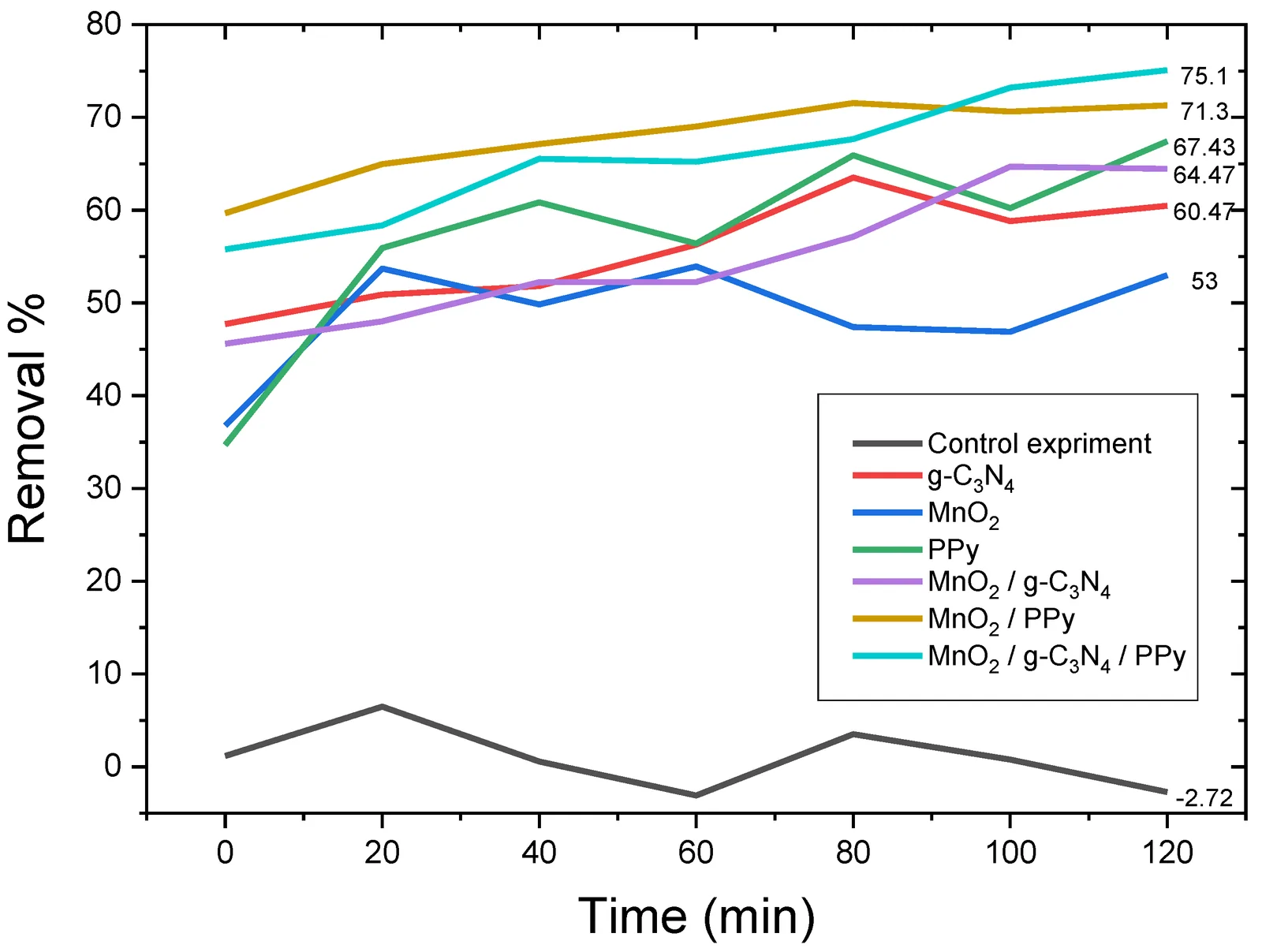
The Adsorption Performance plot (removal % vs. time).

Figure 15 compares the Congo Red removal (%) of the prepared adsorbents and the no-adsorbent control over approximately 0.5–120 min under the same operating conditions. The profiles are used to compare the time-dependent removal behavior of the prepared materials and are not intended as a formal kinetic analysis. As described in Section 2.7, the first plotted point represents the earliest post-contact sample, collected approximately 0.5 min after adsorbent addition and brief mixing. The no-adsorbent control underwent the same sampling, filtration, dilution, and UV–Vis measurement procedure as the adsorption samples. Its apparent removal fluctuated around the initial baseline and reached −2.72% at 120 min, without a sustained decrease in Congo Red concentration. These fluctuations were smaller than the removal observed with the adsorbents, supporting that adsorbent addition was responsible for most of the measured Congo Red removal. In the adsorbent-containing experiments, substantial Congo Red removal was already observed at the first post-contact measurement for all adsorbents, indicating that a considerable fraction of dye uptake occurred rapidly after contact with the materials. Rapid initial adsorption is commonly associated with the availability of readily accessible adsorption sites at the beginning of the process [5,40]. Among the tested samples, MnO_2_/PPy exhibited the highest removal at the first post-contact measurement, at approximately 60%, while the ternary MnO_2_/g-C_3_N_4_/PPy composite showed approximately 56%. The individual materials displayed different early responses, with PPy and MnO_2_ showing higher initial removal than g-C_3_N_4_. Between 20 and 80 min, the relative ranking of the adsorbents changed with contact time. MnO_2_/PPy maintained the highest removal during most of this interval and reached approximately 71% at 80 min, while the ternary MnO_2_/g-C_3_N_4_/PPy composite increased more gradually and remained close to it. The other materials showed more variable time-dependent behavior, particularly MnO_2_, which decreased after its initial increase before partially recovering at longer contact times. From 80 to 120 min, the removal achieved by the ternary composite increased further and reached the highest final Congo Red removal of 75.1% at 120 min. MnO_2_/PPy showed the second-highest final removal at 71.3%, followed by PPy at 67.43%. The MnO_2_/g-C_3_N_4_ composite reached 64.47%, while g-C_3_N_4_ and MnO_2_ reached 60.47% and 53.0%, respectively. Therefore, the ternary composite exhibited the highest final removal among the tested materials at 120 min under the applied conditions. This comparative improvement under the tested batch conditions suggests a beneficial combined effect of the three components on the final adsorption performance. The changes in relative ranking with contact time may reflect differences in the rates at which accessible adsorption sites become occupied and in the time-dependent interactions between Congo Red and the different material surfaces. However, because the individual and binary adsorbent profiles were obtained from single comparative runs, part of the point-to-point variation cannot be clearly distinguished from experimental and analytical variability. The present data therefore do not allow these differences to be assigned to a specific adsorption mechanism. Accordingly, the ternary composite is described as exhibiting the highest final removal at the selected 120 min contact time rather than as being consistently superior throughout the entire experiment.

### 3.7. FTIR Analysis Before and After Adsorption

**Figure 16:**
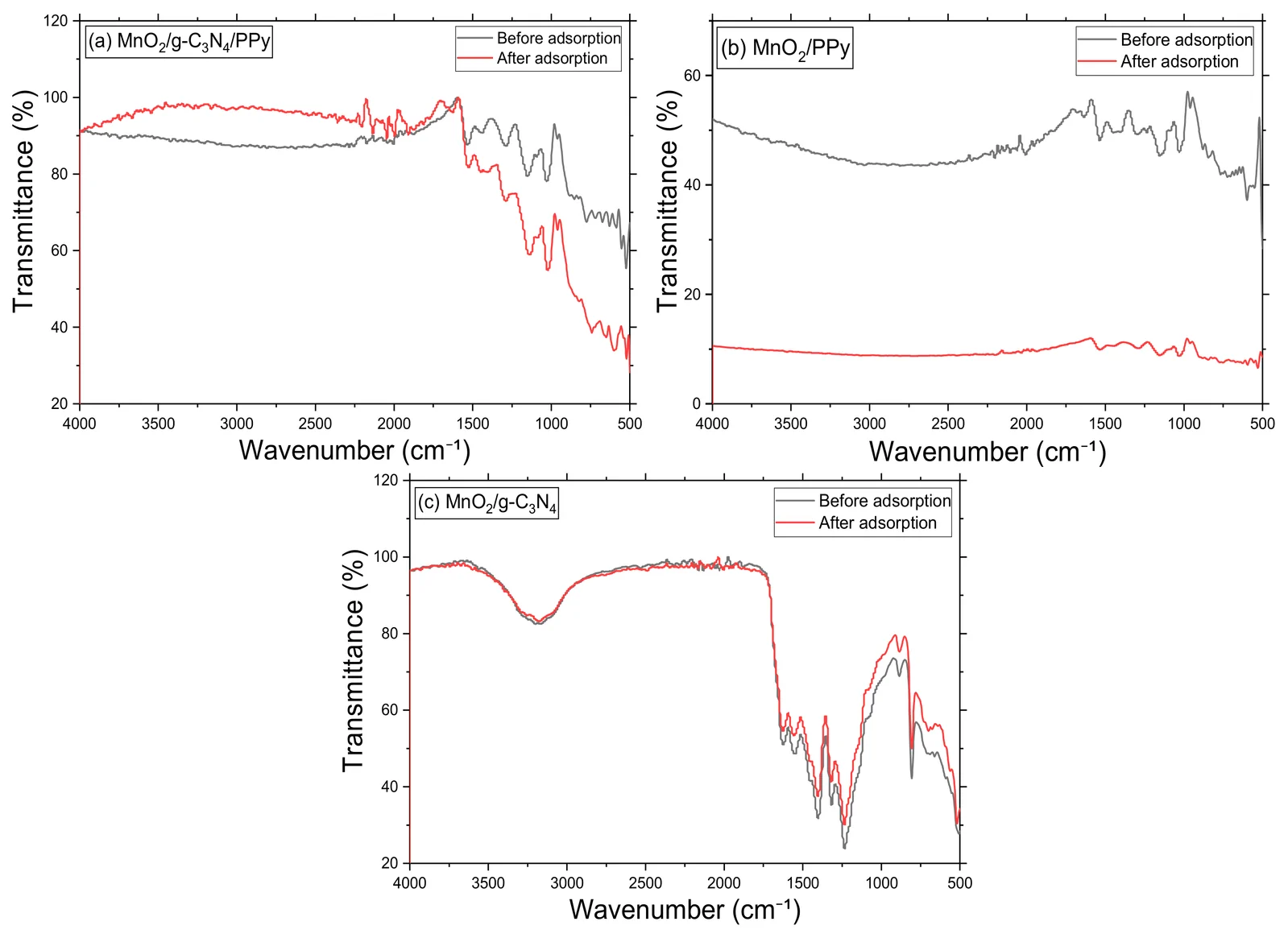
ATR-FTIR spectra of the prepared composites before and after Congo Red adsorption: (**a**) MnO_2_/g-C_3_N_4_/PPy, (**b**) MnO_2_/PPy, and (**c**) MnO_2_/g-C_3_N_4_.

Figure 16 compares the ATR-FTIR transmittance spectra of the MnO_2_/g-C_3_N_4_/PPy, MnO_2_/g-C_3_N_4_, and MnO_2_/PPy composites before and after Congo Red adsorption. The characteristic bands of the three composites were generally retained after adsorption, indicating preservation of their main vibrational features. Because absolute transmittance levels in ATR measurements may be influenced by sample contact and preparation, the comparison was based primarily on band positions and spectral-profile changes rather than absolute transmittance intensity. For MnO_2_/g-C_3_N_4_, the main bands at approximately 1624, 1549, 1402, 1315, 1234, and 806 cm^−1^ remained at nearly the same positions after adsorption. Similarly, the principal bands of MnO_2_/PPy and MnO_2_/g-C_3_N_4_/PPy were largely preserved, although some differences in the spectral profiles were observed within the fingerprint region. For the ternary composite, spectral differences after adsorption were evident particularly in the regions around 1600–1500, 1200–1000, and 800–700 cm^−1^, which overlap with characteristic vibrational regions reported for Congo Red, including aromatic/azo- and sulfonate-related bands [41]. These adsorption-associated spectral changes, particularly within regions characteristic of Congo Red, provide spectroscopic support for the uptake of Congo Red by the composites and complement the concentration-based adsorption results. However, because several Congo Red bands overlap with those of PPy and g-C_3_N_4_, FTIR alone is not sufficient to assign a specific adsorption interaction such as π–π interactions, hydrogen bonding, or electrostatic attraction. In addition, small changes in band position below the instrumental resolution of 4 cm^−1^ were not interpreted as significant chemical shifts.

### 3.8. Observed Adsorption Capacity and Literature Comparison

Based on the end-point removal values, the observed adsorption capacity at 120 min $q_{120}$ was calculated for each prepared material using the relation described in Section 2.8. The calculated values are presented in Table 5. Because all six adsorbents were evaluated under identical experimental conditions, these values provide a direct internal comparison of Congo Red uptake among the prepared materials.

The ternary MnO_2_/g-C_3_N_4_/PPy composite exhibited the highest observed capacity among the prepared materials, reaching 150.20 mg/g at 120 min, followed by MnO_2_/PPy at 142.60 mg/g and PPy at 134.86 mg/g. The g-C_3_N_4_/MnO_2_ composite, g-C_3_N_4_, and MnO_2_ exhibited $q_{120}$ values of 128.94, 120.94, and 106.00 mg/g, respectively. This order is consistent with the final removal-efficiency results and shows that the ternary composite provided the highest Congo Red uptake among the materials tested under identical conditions. The $q_{120}$ values reported in the present study represent fixed-time adsorption capacities and should not be interpreted as equilibrium or maximum adsorption capacities, because adsorption equilibrium was not established across multiple initial Congo Red concentrations. Therefore, comparison with literature values requires consideration of both the experimental conditions and the basis used to determine the reported adsorption capacity.

Table 6 shows that reported Congo Red adsorption capacities vary considerably among composite adsorbents and also depend on how the adsorption capacity is determined. Among studies reporting experimentally measured or time-specific values, PANI@MoS_2_ reached 70.921 mg/g at 120 min [42], Fe_3_O_4_/PPy/C exhibited an experimental adsorption capacity of 227.7 mg/g at 180 min [43], and the LDH/gellan-gum NC-5% composite showed a maximum experimental $q_{e}$ of 99.9 mg/g within the investigated concentration range [44]. In comparison, the MnO_2_/g-C_3_N_4_/PPy composite in the present study exhibited an observed $q_{120}$ of 150.20 mg/g. Thus, the present value falls within the range of experimentally reported Congo Red adsorption capacities represented in Table 6. However, these values should not be interpreted as a direct performance ranking because the initial concentration, adsorbent dosage, pH, temperature, and contact time differ among the studies. Other related composite adsorbents have been evaluated using equilibrium-isotherm analysis or reported maximum adsorption capacities. A maximum adsorption capacity of 68.03 mg/g was obtained for SMNPs/CA/g-C_3_N_4_NS using the Langmuir model [45], while CYP–PPy showed a Langmuir maximum adsorption capacity of 86.66 mg/g [37]. Higher reported maximum capacities include 324.64 mg/g for CA/PPy [46] and a Langmuir-derived $q_{max}$ of 339.8 mg/g for La–NiO–g-C_3_N_4_ [47]. These values span a wide range and were obtained using equilibrium or model-based evaluations rather than the fixed-time approach used in the present study. Accordingly, the value of 150.20 mg/g reported here is used only to describe the observed adsorption performance at 120 min under the applied conditions and is not presented as an equilibrium or maximum adsorption capacity.

### 3.9. Repeatability of the Ternary Composite

**Figure 17:**
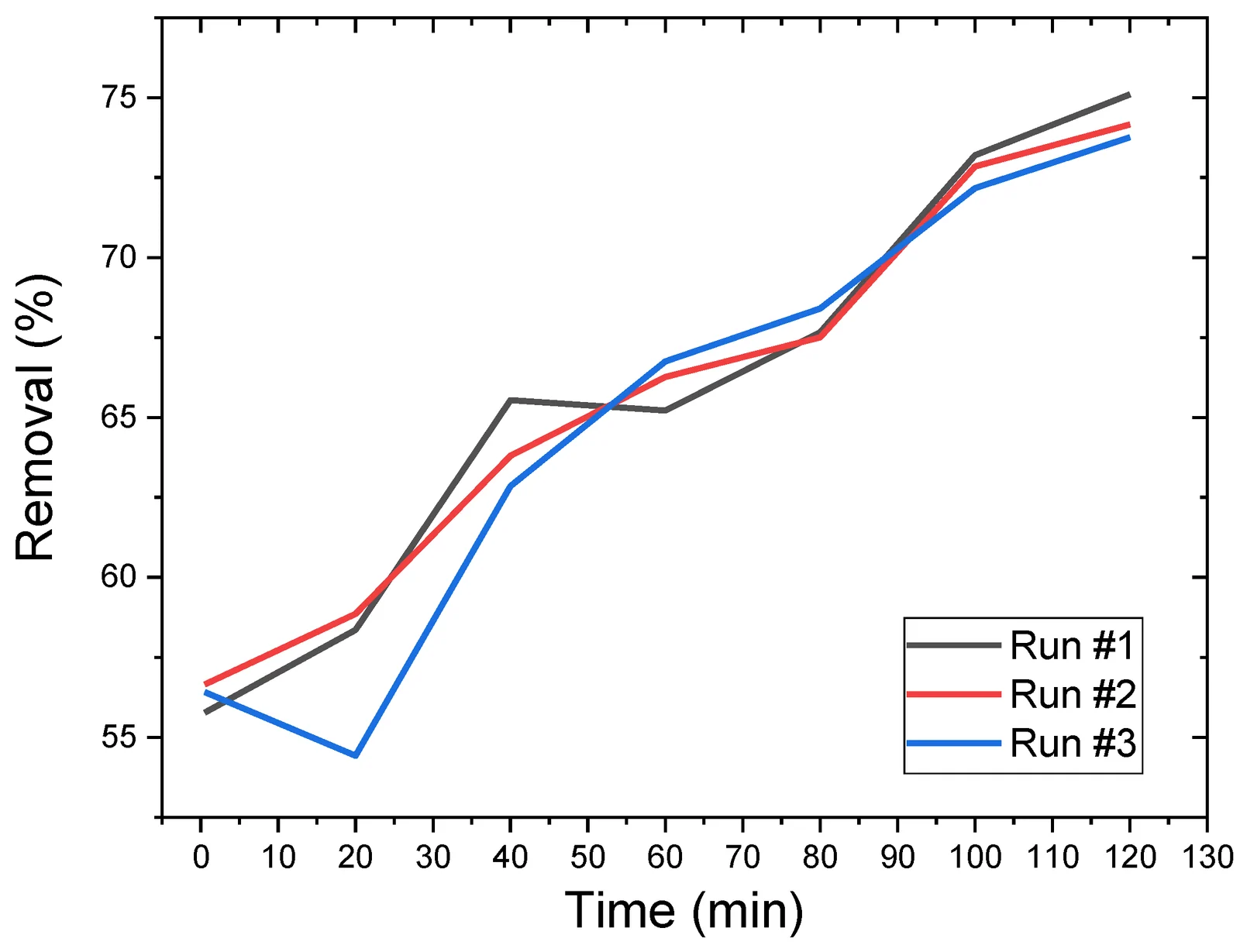
Repeatability test for Congo Red removal using the MnO_2_/g-C_3_N_4_/PPy composite over three independent runs.

To evaluate the repeatability of the adsorption performance, the Congo Red removal experiment was repeated independently three times using the MnO_2_/g-C_3_N_4_/PPy ternary composite. As shown in Figure 17, all three runs showed a similar overall increase in Congo Red removal with contact time. At the first post-contact measurement, collected after approximately 0.5 min, the removal values were within the mid-50% range. Some differences between the runs were observed at intermediate contact times, particularly during the early and middle stages of the experiment; however, the removal values became closer at longer contact times. After 120 min, all three independent runs reached approximately 74–75% removal. These results indicate that, despite some time-dependent variation, the final adsorption performance of the ternary composite was reasonably reproducible under the applied experimental conditions. These independent runs assess repeatability only and do not establish regeneration or cycling stability. Adsorption–desorption cycling, component leaching, and the effects of coexisting ions were not evaluated and remain necessary for assessing practical applicability.

## 4. Conclusions

This study prepared and evaluated MnO_2_, g-C_3_N_4_, PPy, the MnO_2_/g-C_3_N_4_ and MnO_2_/PPy binary composites, and the ternary MnO_2_/g-C_3_N_4_/PPy composite for Congo Red adsorption from water. All materials were compared using the same nominal initial concentration and adsorbent dosage, mixing rate, and contact time under ambient laboratory conditions to allow direct comparison of their adsorption performance. The XRD and SEM/EDS results supported the presence of the expected structural, morphological, and elemental features of the prepared materials; survey XPS characterized all prepared materials, while high-resolution C 1s, N 1s, and O 1s analysis was limited to the ternary composite. Nitrogen adsorption–desorption analysis showed that the main pore-size contributions of the investigated composites occurred within the mesopore range. MnO_2_/g-C_3_N_4_ exhibited the highest BET surface area of 24.203 m^2^ g^−1^, whereas the ternary composite showed an intermediate value of 19.66 m^2^ g^−1^. However, the order of Congo Red removal did not follow the order of BET surface area or pore volume, indicating that these textural parameters alone did not control the adsorption performance. The FTIR spectra recorded before and after adsorption showed adsorption-associated changes that supported Congo Red uptake, although the overlapping bands did not allow a specific adsorption mechanism to be confirmed. The UV–Vis and PL results were considered supporting characterization only and were not interpreted as evidence of photocatalytic activity. Under the applied conditions, the ternary MnO_2_/g-C_3_N_4_/PPy composite achieved the highest final Congo Red removal of 75.1% at 120 min, followed by MnO_2_/PPy at 71.3%. The ternary composite also exhibited the highest observed adsorption capacity at 120 min, with q_120_ = 150.20 mg g^−1^. This value represents a fixed-time adsorption capacity and should not be interpreted as an equilibrium or maximum adsorption capacity. Three independent experiments produced final removal values of approximately 74–75%, supporting the repeatability of the ternary composite result. Overall, the findings show that factors beyond surface area, including the chemical nature and accessibility of the adsorption sites, may have contributed to the observed performance. Future work should evaluate the multi-cycle reusability and operational stability of the ternary composite under realistic wastewater matrices to assess its practical applicability.

## Figures and Tables

**Table 1:** XRD-derived apparent crystallite size, microstrain, and dislocation density values for samples exhibiting defined crystalline diffraction peaks.

Material	Apparent Crystallite Size D (nm)	Microstrain ε (Dimensionless)	Dislocation Density δ (nm^−2^)
g-C_3_N_4_	3.8148	0.0587	0.0687
MnO_2_	21.7001	0.0077	0.0078
g-C_3_N_4_/MnO_2_	14.2132	0.0097	0.0096

**Table 2:** Surface atomic percentages of the elements detected in the XPS survey spectra of the prepared samples.

Material	C (at.%)	N (at.%)	O (at.%)	Cl (at.%)	Mn (at.%)
g-C_3_N_4_	43.9	56.1	—	—	—
MnO_2_	31.6	—	54.8	—	13.6
PPy	71.7	11.7	14.7	1.9	—
MnO_2_/g-C_3_N_4_	38.3	32.7	22.7	—	6.3
MnO_2_/PPy	70.2	19.6	8.1	1.4	0.7
MnO_2_/g-C_3_N_4_/PPy	57.8	28.0	12.5	not quantified	1.6

**Table 3:** High-resolution XPS peak-fitting parameters and assignments for the MnO_2_/g-C_3_N_4_/PPy ternary composite.

XPS Region	Binding Energy (eV)	FWHM (eV)	Relative Area (%)	Literature-Supported Assignment
C 1s	284.80	1.79	82.01	C–C/C=C reference carbon [30]
C 1s	286.47	0.82	4.57	C–N/C–NH_x_ [30]
C 1s	288.70	0.95	8.08	sp^2^ carbon in N–C=N environments of g-C_3_N_4_ [30]
C 1s	290.82	1.54	5.35	π–π* shake-up satellite [31]
N 1s	398.24	0.51	1.53	sp^2^ C=N–C and/or imine-like N [30,32]
N 1s	399.96	1.71	17.71	overlapping bridging N [N–(C)_3_] and neutral pyrrolic N–H environments [30,32]
N 1s	401.68	2.71	80.77	higher-BE N–H/amino and/or positively charged PPy-N environments [30,32]
O 1s	530.78	2.62	14.39	low-BE oxygen contribution; Mn–O/Mn–OH and oxygenated-carbon environments may overlap [31,33]
O 1s	532.08	1.44	51.55	surface/adsorbed oxygen and oxygenated-carbon environments [31,33]
O 1s	533.47	1.40	34.07	higher-BE surface hydroxyl-containing oxygen species [33]

**Table 4:** BET- and NLDFT-derived textural properties of the investigated binary and ternary composites.

Material	BET Surface Area (m^2^ g^−1^)	NLDFT Surface Area (m^2^ g^−1^)	NLDFT Pore Volume (cm^3^ g^−1^)	NLDFT Modal Pore Width (nm)
MnO_2_/PPy	12.39	8.370	0.019	2.647
MnO_2_/g-C_3_N_4_	24.203	17.036	0.034	2.769
MnO_2_/g-C_3_N_4_/PPy	19.66	14.045	0.031	2.647

**Table 5:** Observed adsorption capacity at 120 min ($q_{120}$) for the prepared adsorbents.

Sample	Observed Adsorption Capacity at 120 min $(q_{120},mg/g).$
MnO_2_/g-C_3_N_4_/PPy (ternary)	150.20
MnO_2_/PPy (binary)	142.60
PPy (single)	134.86
MnO_2_/g-C_3_N_4_ (binary)	128.94
g-C_3_N_4_ (single)	120.94
MnO_2_ (single)	106.00

**Table 6:** Comparison of Congo Red adsorption capacities reported for selected composites and related adsorbents.

Adsorbent	Reported Capacity (mg/g)	Capacity Basis	Relevant Reported Conditions
MnO_2_/g-C_3_N_4_/PPy (this work)	150.20	Observed $q_{120}$	$C_{0}$ = 200 mg/L; dose = 1.0 g/L; 120 min; room temperature; unadjusted pH
PANI@MoS_2_ [42]	70.921	Highest reported adsorption at 120 min	pH 5; 120 min; 50 °C
Fe_3_O_4_/PPy/C [43]	227.7	Experimental adsorption capacity	$C_{0}$ = 120 mg/L; dose = 0.5 g/L; pH 7; 180 min
LDH/gellan-gum NC-5% [44]	99.9	Maximum experimental $q_{e}$ in the investigated concentration series	$C_{0}$ = 10–300 mg/L; 10 mg adsorbent; pH 5; 180 min; 298 K
SMNPs/CA/g-C_3_N_4_NS [45]	68.03	Langmuir $q_{max}$	pH 5; 120 min
CYP–PPy [37]	86.66	Langmuir $q_{max}$	Equilibrium-isotherm analysis
CA/PPy [46]	324.64	Reported maximum adsorption capacity	pH 6.5; equilibrium reached at 180 min
La–NiO–g-C_3_N_4_ [47]	339.8	Langmuir-derived $q_{max}$	Freundlich provided the better overall isotherm fit

## Data Availability

The original contributions presented in this study are included in the article. Further inquiries can be directed to the corresponding authors.
